# Bridging Pediatric to Adult Care: A Scoping Review on Transitional Care for Individuals with Congenital Heart Disease Using Data Mining Techniques to Identify Key Topics

**DOI:** 10.1007/s11886-026-02377-1

**Published:** 2026-05-22

**Authors:** Salvatore Angileri, Rocco Mazzotta, Daniele Ciofi, Rosario Caruso, Silvia Favilli, Gaia Spaziani, Gianluca Conte, Serena Francesca Flocco, Jeroen M. Hendriks, Giulia Maga, Pier Mario Perrone, Silvana Castaldi, Edward Callus, Massimo Chessa, Arianna Magon, Maddalena De Maria

**Affiliations:** 1 Department of Biomedicine and Prevention, University of Rome Tor Vergata, Rome, Italy; 2 Department of Health Care Professions Unit, Meyer Children’s Hospital – IRCCS, Florence, Italy; 3 Health Professions Research and Evidence Transfer Unit, IRCCS MultiMedica, Sesto San Giovanni, Italy; 4 Department of Biomedical Sciences for Health, University of Milan, Milan, Italy; 5 Pediatric and Transition Cardiology, Meyer Children’s Hospital – IRCCS, Florence, Italy; 6 Department of Medicine and Surgery, Kore University of Enna, Enna, Italy; 7 Department of Nursing, Maastricht University Medical Centre, Maastricht, The Netherlands; 8 Department of Health Services Research, Care and Public Health Research Institute, Maastricht University, Maastricht, The Netherlands; 9 Centre for Heart Rhythm Disorders, University of Adelaide, Adelaide, Australia; 10 Department of Public Health, Experimental and Forensic Medicine, Section of Hygiene, University of Pavia, Pavia, Italy; 11 Fondazione IRCCS Ca’ Granda Ospedale Maggiore Policlinico, Milan, Italy; 12 Clinical Psychology Service, IRCCS Policlinico San Donato, San Donato Milanese, Milan, Italy; 13 Adult Congenital Heart Disease (ACHD) Unit, IRCCS Policlinico San Donato, San Donato Milanese, Milan, Italy; 14 Faculty of Medicine, Vita-Salute San Raffaele University, Milan, Italy; 15 Department of Life Health Sciences and Health Professions, Link Campus University, Rome, Italy

**Keywords:** Congenital heart disease, Transitional care, Adolescents, Young adults, Scoping review, Topic modeling

## Abstract

**Background:**

Adolescents and young adults with congenital heart disease (CHD) face significant challenges when transitioning from pediatric to adult care. Despite growing recognition of its importance, transitional care remains inconsistently implemented across healthcare systems, and a summary of published literature in this regard is still missing. This scoping review aims to systematically map the literature on CHD transitional care and identify key topics and trends.

**Methods:**

Following Joanna Briggs Institute guidelines, we conducted a comprehensive search across seven electronic databases using the Population, Concept, and Context framework. A total of 73 studies were included. Data were extracted and analyzed using Latent Dirichlet Allocation to identify core topics, and Multiple Correspondence Analysis was applied to explore thematic relationships and validate topic structure. Lexicometric analysis assessed the linguistic complexity and specificity of the literature.

**Results:**

Three major themes emerged: (1) Education, Self-Management, and Structured Support, (2) Timing, Knowledge Transfer, and Developmental Needs, and (3) Transition Program Implementation and Coordination. These themes reflect an increasing focus on structured educational strategies, developmentally tailored care, and system-level program delivery. Education-focused interventions were more frequently found in recent, high-quality experimental studies. In contrast, studies addressing timing and developmental needs and those focused on implementation were more common in earlier-phase or heterogeneous research contexts.

**Conclusion:**

Transitional care for individuals with CHD requires more standardized, evidence-based approaches. Improved documentation when reporting transitional care is essential to enhance fidelity, scalability, and long-term impact. This review provides a foundation for developing outcome-focused research and supports designing individualized, high-quality transition programs.

**Supplementary Information:**

The online version contains supplementary material available at 10.1007/s11886-026-02377-1.

## Introduction

Congenital heart disease (CHD) is one of the most common birth defects worldwide, with an estimated birth prevalence of 1,787.6 cases per 100,000 newborns, a slight increase from previous decades [1]. Advances in pediatric cardiology, including enhanced diagnostic and surgical techniques, have contributed to a significant decline in CHD-related mortality, with global CHD deaths falling by 34.5% between 1990 and 2017 [1]. As a result, more adolescents and young adults with CHD are surviving into adulthood, with approximately 11.99 million individuals living with CHD worldwide [1]. In the current context, this growing population faces unique lifelong challenges, requiring complex, ongoing healthcare and psychosocial support to effectively manage their condition and associated health risks [2].

Transition clinic is a structured, multi-dimensional process to prepare adolescents and young adults with CHD to move from pediatric to adult healthcare services [3]. Transitional care for adolescents with CHD involves structured interventions that help patients acquire the skills and knowledge necessary for independent health management, which is essential for ensuring continuity and quality of care as they move from pediatric to adult healthcare settings [2]. Specifically, transitional care aims to enhance health literacy, empowerment, self-management, and adherence to medical recommendations, addressing significant gaps between pediatric and adult healthcare services [4]. Without such support, these young patients face elevated risks, including non-adherence to treatment, increased morbidity, and interruptions in cardiac follow-up [5]. Research highlights that well-executed transitional care, which often includes a dedicated transition coordinator or joint pediatric-adult clinics, is linked to improved self-management, health outcomes, and patient empowerment [4, 5]. For these reasons, international consensus statements emphasize the need for structured transition care programs tailored to the unique needs of adolescents with CHD, which can significantly improve long-term health outcomes and quality of life [2].

Transition involves a comprehensive, multifaceted process addressing the clinical, psychosocial, and educational needs of these patients and their families as they gradually prepare to manage their health autonomously [2, 4, 5]. Transition programs for adolescents with CHD typically begin in early adolescence, often around age 12, allowing ample time for patients to build the skills needed for independent health management [6]. These programs are usually organized around structured interventions, including health education, self-management training, and psychosocial support [3, 5]. Many programs are led by multidisciplinary teams, often including a transition coordinator, nurses, cardiologists, and sometimes psychologists, who collaboratively guide patients and families through the phases of transition. A successful transition program may also involve joint or overlapping appointments with pediatric and adult care providers, creating a familiar and supportive bridge as patients prepare for transfer. Programs often conclude with a comprehensive transfer plan that ensures continuity by clearly defining roles and responsibilities within the adult healthcare setting. Therefore, the transition is distinct from the concept of transfer, which is the formal event where responsibility for care shifts from pediatric to adult providers, ideally within an adult CHD center [3]. While transition spans several years and adapts to each patient’s developmental readiness, transfer marks the endpoint of this preparatory phase, occurring at a predefined or flexible age based on institutional protocols [3].

Despite its importance, gaps persist in the implementation of transitional care for patients with CHD [6]. These gaps include the lack of standardized guidelines, fragmented approaches across institutions, and limited attention to patients’ psychosocial and cognitive needs. Furthermore, the growing body of literature on this topic makes it challenging to synthesize existing evidence and translate it into practical guidelines [6–9]. Given this vast and complex body of literature, a comprehensive review is essential to map the current landscape and identify emerging trends, as it may allow researchers to organize key topics in CHD transitional care [10]. Such a review is necessary to systematically capture the breadth of research, summarize existing evidence by highlighting emerging topics and guiding future research, and support the development of standardized, evidence-based practices in transitional care for patients with CHD. For these reasons, this scoping review aims to systematically map the existing literature, describe the emerging topics underlying the published literature, and explore literature trends between topics and characteristics of the available literature, such as publication year, country of origin, type of publication, and the quality of the reporting regarding the implementation of transitional care interventions.

## Methods

### Design

We conducted a scoping review to address the research question: “What are the key topics regarding transitional care for adolescents and young adults with CHD and the emerging trends between topics and characteristics of the available literature?” To guide our search strategy, we employed the *population*,* concept*,* and context* (PCC) criteria to design the main research question and database-specific queries [11, 12].

The Population included adolescents and young adults with CHD, focusing on those transitioning from pediatric to adult healthcare. Whilst we did not specify an exact age range, it is typically recognized that the transition process commences around the age of 12. The Concept centered on transitional care, specifically the processes, interventions, and outcomes associated with managing the shift from pediatric-focused to adult-focused care for CHD patients. The Context covered healthcare settings, policies, and practices involved in providing transitional care for CHD patients across diverse clinical and geographical environments.

This scoping review adheres to the Joanna Briggs Institute (JBI) guidelines for scoping review methodology [11, 12]. Additionally, the Preferred Reporting Items for Systematic Reviews and Meta-Analyses extension for Scoping Reviews (PRISMA-ScR) was utilized to structure both the reporting of the research process and the presentation of results [13].

### Eligibility Criteria

The following eligibility criteria guided study selection: publications were included without time limits, available in either English or Italian, or any language when an HTML version of the paper was accessible, allowing for translation into English using web-based translation applications. Eligible studies focused on transitional care for adolescents and young adults with CHD without specifying a precise age range to improve the sensitivity of the review, and consistent with the definition of transition that is intended as a process that begins in early adolescence, with variation regarding when the process is activated, and lasts up until adulthood. There were no restrictions on study design or country of origin. However, studies were excluded if they did not explore transitional care with empirical data or policy analysis relevant to CHD.

### Operational Definition of Transition

In this review, transition is defined as a structured, multi-dimensional process aimed at preparing adolescents and young adults with CHD to move from pediatric to adult healthcare services [3]. Transition encompasses not only the transfer of medical care but also the development of empowerment, self-management skills, health literacy, and psychosocial readiness to adapt to the adult healthcare environment. Transition is understood as a longitudinal process that ideally begins in early adolescence and continues until the individual is fully integrated into adult healthcare services, ensuring continuity and quality of care across the lifespan.

### Information Sources

The information sources for this scoping review included seven databases and registers to ensure a comprehensive and thorough literature search. Records were identified from PubMed, Embase, Cochrane Review, Cochrane Trial, PsycINFO, CINAHL, and ClinicalTrials.gov, providing a wide array of research and clinical studies relevant to transitional care for CHD patients. Additionally, citation searching was conducted to capture any pertinent further studies not identified in the initial database searches.

### Search Strategy

Our search strategy was designed in consultation with an expert librarian to effectively capture the PCC criteria, as described before, central to our research question. This approach aimed to balance sensitivity, ensuring that relevant studies on transitional care for adolescents and young adults with CHD were included, with specificity, to avoid an overabundance of irrelevant results. The strategy included a comprehensive set of search terms and Boolean operators across multiple databases, carefully constructed to capture variations in terminology related to CHD, transition, and the target age groups. Searches were conducted in major databases, including PubMed, Embase, CINAHL, PsycINFO, and the Cochrane Library, and were supplemented by citation searches to ensure broad coverage. The last search was completed on November 7, 2024. The detailed search queries for each database are provided in Supplementary File 1.

### Selection Process

The study selection process began with organizing records sourced from electronic databases using Rayyan software, while additional studies identified through hand-searching were manually incorporated and subjected to the same selection criteria [14]. Initially, duplicate entries from the electronic databases were removed to ensure a streamlined selection.

In the screening phase, two authors independently evaluated the studies based on their titles and abstracts, following a double-blind approach during the first round. Any discrepancies between the authors were addressed through consensus discussions in a subsequent round of screening. Studies that met the pre-established eligibility criteria were then reviewed in full text to confirm their inclusion in the scoping review. To further ensure consistency in the selection process, a third author independently assessed the studies ultimately included. The entire study selection procedure adhered to the PRISMA-ScR guidelines to maintain transparency and rigor throughout the process.

### Data Extraction

Data were initially extracted using a standardized extraction form capturing key details: authors and year, type of publication, country, main objective, study design, sample, transition characteristics, and key findings. This initial data extraction focused on creating a comprehensive and systematic summary of each study. The Key Findings field involved extensive reporting of the results, discussion, and interpretation sections from each study, creating a detailed textual corpus based on the extracted information.

When feasible, for primary studies implementing a transition model, the Transition Intervention Description and Replication (TIDieR) checklist, an extension of the Consolidated Standards of Reporting Trials (CONSORT) 2010 and the Standard Protocol Items: Recommendations for Interventional Trials (SPIRIT) 2013 reporting guidelines, was applied to evaluate the quality of reporting and the replicability of interventions [15]. Using the TIDER checklist, we assessed each transition model’s reporting completeness, computing a final completeness rate from 0 to 100%.

To ensure the textual corpus was suitable for topic modeling analytics, we implemented several quality control measures. We focused on capturing detailed and contextually rich segments from each study’s findings, carefully preserving the nuances in reporting. Redundant or extraneous information was minimized to maintain consistency across entries, allowing for clearer interpretation in the modeling process. Additionally, we standardized terminology and checked for consistency in phrasing across studies to enhance the comparability of topics identified during analysis. These steps aimed to ensure a reliable and high-quality textual corpus that could accurately support topic modeling and reveal patterns and themes within the literature on transitional care for CHD.

### Data Analysis

The data analysis process for this scoping review was conducted in accordance with the JBI manual [16]. In addition, we introduced a novel method to enhance traditional scoping review analysis by incorporating topic modeling analytics, a technique increasingly applied in recent literature to streamline the identification of themes and trends within large textual corpora [10].

Instead of manually interpreting the extracted information, we leveraged topic modeling to analyze the textual corpus generated from our data extraction. Our workflow began by calculating lexicometric properties of the corpus to understand its structure. These metrics provide foundational insights into the characteristics of the text data, helping to gauge its complexity, variability, and density of information [17]. The total number of texts represents the number of documents in our dataset, giving an overall sense of the data volume we are analyzing. The number of word occurrences is the total count of words (tokens) across all texts, reflecting the dataset’s size and helping to measure linguistic richness. The unique word forms metric counts the distinct words (or types) in the corpus, showing vocabulary diversity; a higher number of unique words suggests a broader range of terms and potentially more nuanced topics. Hapax legomena, or words that appear only once in the entire corpus, often indicate specificity or technicality in the text, as these unique words typically relate to specialized concepts or terminology. The type-token ratio (TTR), the ratio of unique words to total words, indicates lexical diversity. A high TTR implies less repetition and a wider variety of terms in the corpus. Together, these lexicometric properties provide insights into the corpus’s richness and linguistic diversity, setting a solid foundation for subsequent topic modeling by ensuring the corpus is both detailed and varied enough to reveal meaningful themes [17].

We then applied Latent Dirichlet Allocation (LDA), a widely used topic modeling technique, to identify underlying topics within the textual corpus, which was recently applied to scoping reviews [18, 19]. Determining the correct number of topics is essential to ensure that each topic is distinct and meaningful without over- or under-dividing the data. We evaluated each configuration using established metrics that assess the quality of topic modeling solutions [20–23]. Each of these metrics offers a unique perspective on the coherence and distinctiveness of topics within the data. Griffiths, for instance, focuses on the model’s likelihood, maximizing the probability of observing the corpus given the topics, which helps in identifying coherent topics [20]. Cao uses the distance between topics to evaluate how distinct they are, aiming to minimize overlap [22]. Arun analyzes the divergence between the distribution of topics in documents and words across topics, helping to identify a natural fit for topic numbers [23], while Deveaud measures how well topics capture the underlying data structure, balancing coherence with distinctiveness [21]. Bayesian optimization enhanced this process by systematically exploring the configurations and selecting the number of topics that maximized both coherence and separation [24]. Through this iterative approach, we identified the most coherent and representative set of topics for the data, ensuring a reliable and meaningful foundation for interpreting the results.

Once the optimal number of topics was determined, it became feasible to analyze how closely each document related to each topic by calculating the probability that the content of a document belonged to each identified topic [19]. This process involved applying the trained LDA model, which assigned a set of probabilities—one for each topic—to each document in the corpus. These probabilities represent the likelihood that a document belongs to a specific topic based on the words and patterns identified by the model during training. For each document, the model generated a probability distribution across all topics, allowing us to identify the dominant topic for each document, which was simply the topic with the highest probability in that document’s distribution. This dominant topic assignment was then recorded in the dataset, along with the complete set of probabilities for each topic. Additionally, for each identified topic, the top words were visualized using word clouds to provide a more intuitive understanding of the topic content. We explored the probability of each word’s appearance within its topic, helping us to assign meaningful labels to each topic. We identified the 20 most frequent terms for each topic and organized them by probability to determine the most characteristic words. This step helped in labeling the topics based on their dominant terms and thematic coherence, further facilitating the interpretation of the results.

To validate the identified topics, we visualized them in a two-dimensional space using Multiple Correspondence Analysis (MCA) based on key categorical variables from the dataset [25]. The MCA included the following variables: Period of publication (categorized as “Last 5 years,” “6–10 years ago,” and “More than 10 years ago”), Continent of origin (Europe, North America, Asia, Global), Type of publication (journal article or conference proceeding), Study design (e.g., RCT, quasi-experimental, cross-sectional, qualitative), TIDieR completeness score (categorized in quartiles based on the percentage of checklist items reported), and the assigned topic label derived from LDA modeling. This analysis allowed us to explore documents’ spatial distribution and clustering relative to these variables and examine how specific methodological and contextual characteristics aligned with each thematic topic. We aimed to observe distinct spatial groupings, as documents with similar characteristics clustered together, by plotting these variables in the MCA’s two-dimensional space. This clustering validated the coherence of the identified topics and provided visual confirmation of thematic distinctions within the literature on transitional care for patients with CHD. The strength of the association between variables and dimensions derived from MCA was estimated using eta-squared (η²) values and statistical significance was assessed through p-values.

All analyses were conducted in the R programming environment (R Core Team, 2023), version 4.5.0.

### Quality Assessment

Consistent with JBI and PRISMA-ScR guidelines [16], a formal quality assessment was not conducted for this scoping review. Scoping reviews aim to map the breadth of existing literature and identify key themes and gaps rather than critically appraise methodological quality [11]. Given the variety of study designs, applying a uniform quality assessment tool would be challenging and not essential for our objectives. Instead, we report methodological details—such as study design, sample characteristics, and data collection methods—to support transparency and contextualization of findings while adhering to the exploratory nature of this review.

## Results

### Study Selection

**Fig. 1:**
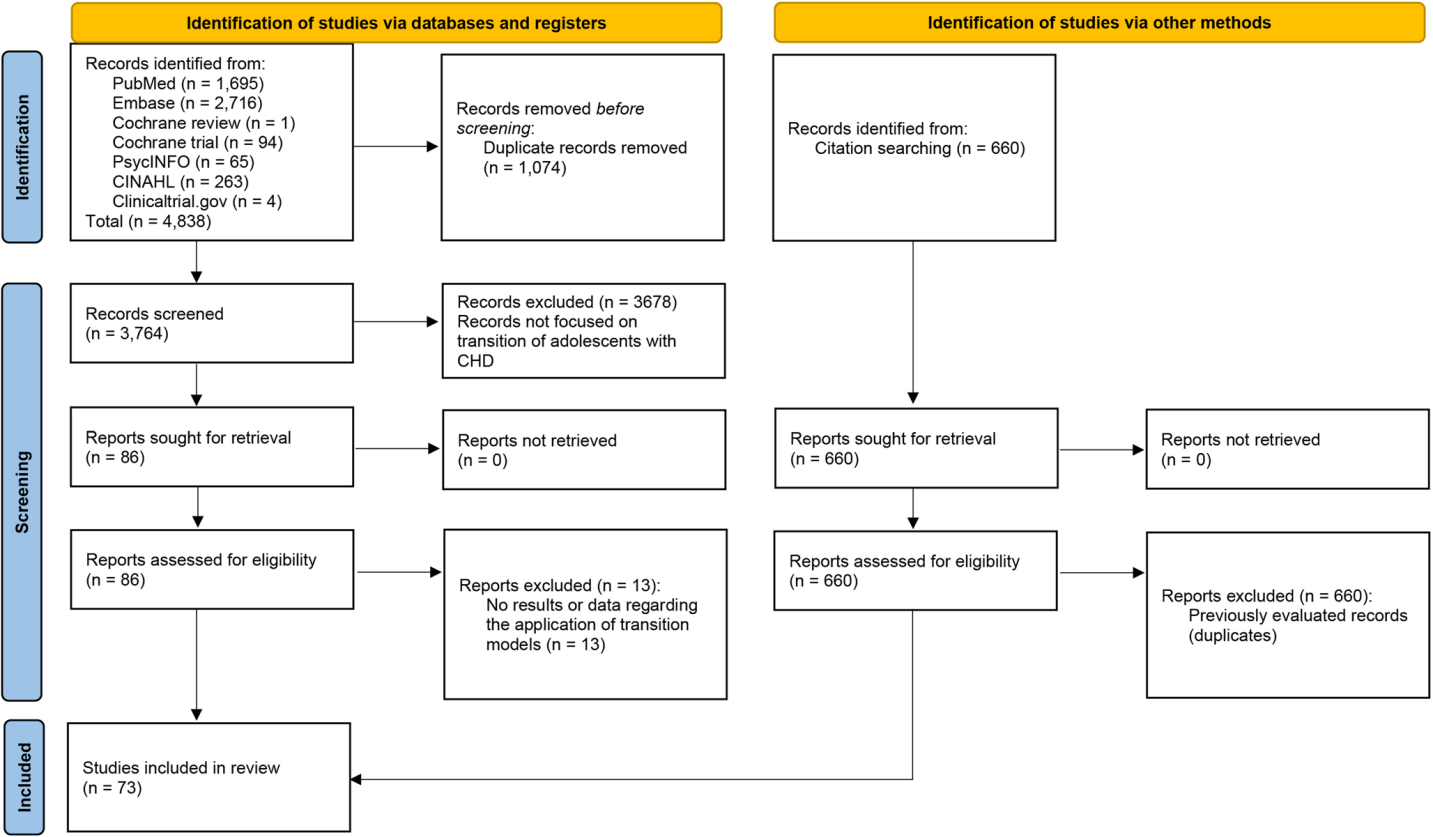
PRISMA flow diagram of study selection

The study selection process is outlined in Fig. 1. Our initial search yielded 4,838 records from various databases, including PubMed, Embase, Cochrane Review, Cochrane Trial, PsycINFO, CINAHL, and ClinicalTrials.gov. After removing 1,074 duplicate records, 3,764 were screened based on titles and abstracts, excluding 3,678 records that did not focus on the transition of adolescents and young adults with CHD. We sought retrieval for 86 reports, all of which were successfully accessed. Upon further assessment, 13 reports were excluded for not focusing on CHD.

An additional 660 records were identified through citation searching, but all were found to be previously evaluated duplicates and were thus excluded. Ultimately, 73 studies met the eligibility criteria and were included in the scoping review [26–93].

### Study Characteristics

The included studies in this review comprise a total of 73 publications [26–93], with 68 journal articles (93.1%) [26–71, 73–86, 88, 92, 93] and five conference proceedings (22.7%) [72, 87, 89–91]. The 68 journal articles included in this review encompass a range of study designs. There were 14 randomized controlled trials (RCTs) [39, 59, 66, 67, 69, 72, 77, 85, 86, 89, 91–93]. Four studies employed a quasi-experimental design [73, 74, 81, 90], two were mixed-methods [49, 84], six were retrospective studies (cohort or case-control) [37, 58, 62, 75, 79, 88], eight articles were cross-sectional studies [6, 38, 45, 76, 78, 80, 83, 87]. In addition, 23 were literature reviews (narrative or systematic) [27–31, 35, 41–43, 47, 53–57, 60, 61, 63–65, 68, 71, 82], four were editorials [9, 26, 50, 52], five were expert consensus [2, 4, 32, 40, 70], five were qualitative research [5, 36, 38, 44, 48], and two were protocols [33, 67]. A detailed breakdown of the characteristics of each study, including study design, country, objectives, and key findings, is provided in Supplementary File 2.

### Quality of the Description of the Transition Models

The median completeness rate of the TIDieR items is 75% (Q1- Q3: 66.67% − 83.33%), with an interquartile range of 16.66%. Studies that reported transition with a high-quality description (TIDieR higher than the 3rd quartile ≥ 83.33%) were 25 [9, 26, 29, 30, 32, 39, 42, 43, 45, 49, 53, 54, 58, 61, 62, 67, 70, 75, 77, 84, 86, 88, 89, 92, 93].

The items with the highest levels of completeness include key aspects such as the description of materials and procedures (what), which refers to the physical materials and procedural steps used in the intervention [72–93]. Information about who provided the intervention (who provided), including the background, training, and expertise of those delivering it, is complete. Additionally, information on the frequency, duration, and intensity of the intervention sessions (when and how much) is not consistently reported, limiting replication [74, 80, 83, 87]. Tailoring, detailing if and how the intervention was personalized or adapted to participant needs, is missing, as are details on any modifications made to the intervention during the study (modifications), including rationale and methods for changes. Finally, reporting on intervention fidelity (how well, both planned and actual), which assesses whether it was delivered as intended and any strategies used to ensure adherence, is frequently overlooked.

### Emerging Topics

#### Lexicometric Characteristics

As shown in Table 1, the corpus analyzed for this review consisted of 73 records, encompassing a total of 4,644 word occurrences. Within this corpus, there were 1,123 unique word forms, indicating the vocabulary diversity used across the texts. Among these, 561 were hapax legomena, or words that appear only once, suggesting a level of specificity in language or specialized terminology used within the field. The mean occurrences of tokens per article (text) was 63.62, highlighting the average density of information across documents. The TTR, which measures lexical diversity, was 24.18%, indicating moderate variability in vocabulary across the corpus, which supported adequacy for topic modeling procedures.

**Table 1:** Lexicometric characteristics of the corpus

Characteristic	Value
Number of texts	73
Total word occurrences (N)	4,644
Unique word forms (V)	1,123
Hapax legomena (H)	561
Mean occurrences per text	63.62
Type-token ratio (V/N)	24.18%

a. Number of texts: Total number of documents included in the corpus.

Total word occurrences (N): The overall count of words (tokens) across all texts, indicating corpus size.

b. Unique word forms (V): The number of distinct words (types) in the corpus, reflecting vocabulary diversity.

c. Hapax legomena (H): Words that appear only once in the corpus, often specific or specialized terms.

d. Mean occurrences per text: The average number of word occurrences per document, showing density of information.

e. Type-token ratio (V/N): The ratio of unique words to total words, measuring lexical diversity within the corpus (in percentage).

#### LDA

**Fig. 2:**
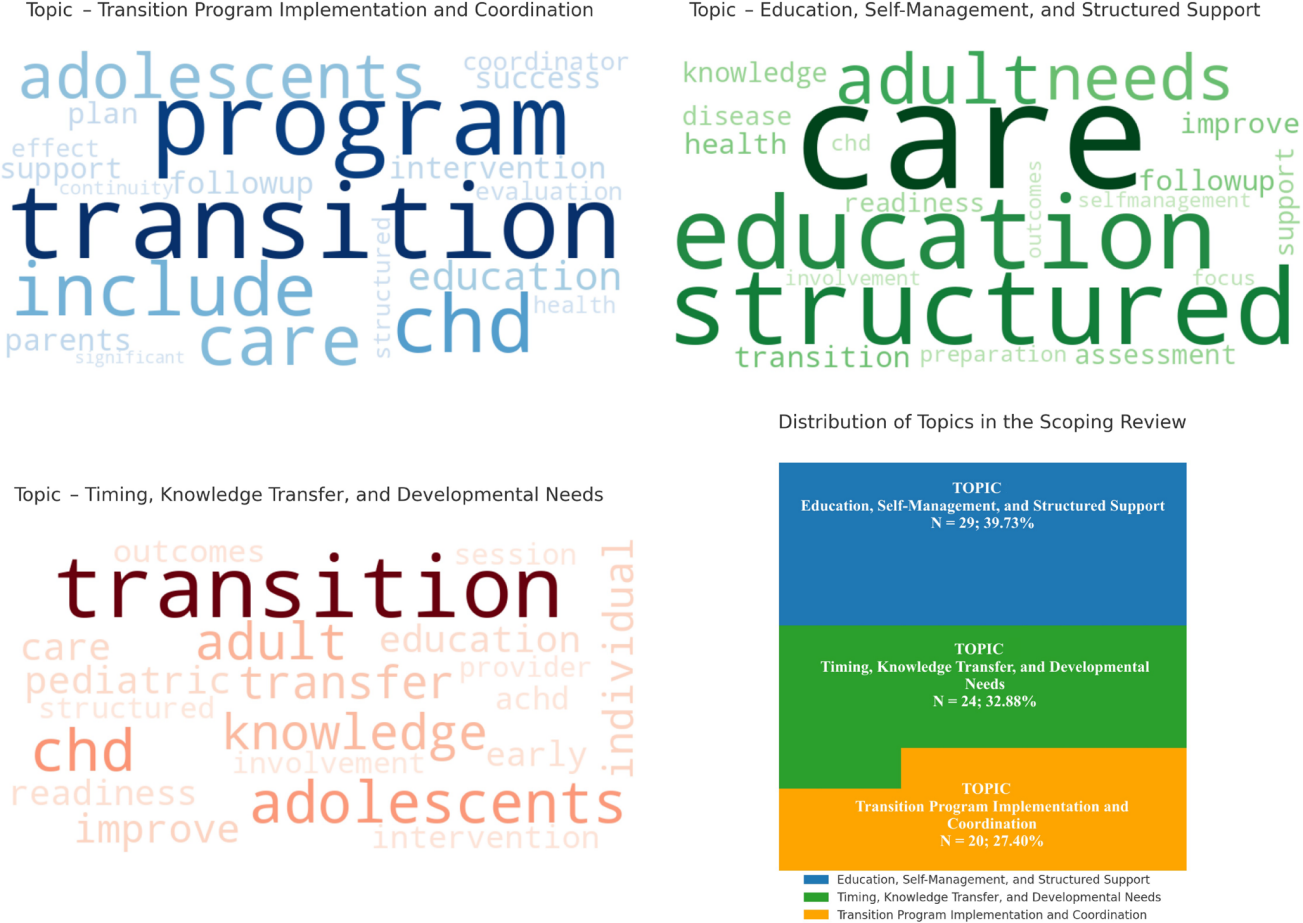
Topic modeling results: word clouds and distribution. Note: The word clouds visually depict the most frequent terms associated with each of the three identified topics based on latent dirichlet allocation. Top Left: Topic 3 – transition program implementation and coordination, Top Right: Topic 1 – education, self-management, and structured support, bottom left: topic 2 – timing, knowledge transfer, and developmental needs; bottom right: proportional distribution of the three topics within the included literature (*N* = 73). Word size reflects the term probability within each topic. The distribution chart highlights the relative prevalence of each theme in the scoping review

The detailed procedure that led to the identification of the emerging topics is provided in Supplementary File 3. After processing the corpus and applying LDA with Bayesian optimization, we identified three distinct topics. These topics and their top associated words are visualized in Fig. 2 and further detailed in Supplementary File 3.

The identified topics are as follows: Topic 1 (*n* = 29; 39.73%) – “Education, Self-Management, and Structured Support” [2, 9, 39, 45, 47, 49, 50, 52, 53, 55–57, 60, 66, 71, 73, 74, 76, 81, 82, 84–86, 88, 89, 91–93], Topic 2 (*n* = 24; 32.88%) – “Timing, Knowledge Transfer, and Developmental Needs” [6, 27, 30, 31, 33, 37, 38, 41–44, 58, 59, 62, 63, 77–80, 83, 87], and Topic 3 (*n* = 20; 27.40%) – “Transition Program Implementation and Coordination” [4, 5, 26, 29, 35, 36, 40, 54, 61, 64, 65, 67, 68, 70, 72, 75, 77, 83, 90].

#### Topic 1 – “Education, Self-Management, and Structured Support”

Topic 1 represents the largest body of literature and focuses on structured interventions that support adolescents and young adults with CHD in acquiring the knowledge and skills needed to manage their condition as they move toward adult care (see detailed information from individual studies in Supplementary File 2). These studies prioritize education, self-management training, and patient empowerment as critical components of effective transitional care [2, 9, 39, 45, 47, 49, 50, 52, 53, 55–57, 60, 66, 71, 73, 74, 76, 81, 82, 84–86, 88, 89, 91–93].

The interventions span the continuum of care and are often structured across three key phases: pre-transition, transition, and post-transition. In the pre-transition phase, which often begins between the ages of 12 and 14 years [39, 47, 85, 91, 92], the main emphasis is assessing and enhancing transition readiness. This includes evaluating developmental maturity and health literacy, commonly using tools such as the Transition Readiness Assessment Questionnaire [84, 86] or the Readiness for Transition Questionnaire [71, 82]. Studies in this phase highlight the importance of early patient engagement and gradual responsibility shifting to foster autonomy [45, 57].

During the transition phase, typically spanning mid-to-late adolescence (ages 14 to 18 years) [82, 89, 91], empowerment emerges as a central outcome [39, 47, 85, 92]. This is often gradually achieved through gains in disease-specific knowledge, self-efficacy, and adherence to medical follow-up. Interventions in this phase include structured educational sessions, peer-supported group activities, and individualized support plans. Programs like STEPSTONES [39, 47, 85, 92] and CHAPTER [67] have demonstrated significant improvements in empowerment using validated instruments such as the Gothenburg Young Persons Empowerment Scale and enhanced self-management and communication skills outcomes.

Post-transition phase outcomes, typically assessed after age 18, once care has been transferred to adult services, are less consistently defined across the literature. Where reported, these outcomes often relate to care continuity, patient satisfaction, or engagement in adult care, such as successful attendance at adult CHD clinics [53, 57, 91]. However, systematic evaluation of post-transition outcomes remains limited, and more longitudinal studies are needed to establish best practices during this phase.

Some authors underscore the critical influence of social determinants of health (SDOH), including race, socioeconomic status, geographic location, and insurance coverage, on the transition from pediatric to adult care in individuals with CHD [71]. Their findings highlight that adolescents from marginalized or underserved backgrounds face greater barriers to successful transfer, increased risk of loss to follow-up, and reduced access to specialized adult care. The authors call for multilevel strategies, including policy reform, bias reduction, and culturally sensitive transition programs, to promote equity and address disparities in transitional care outcomes [71].

Several randomized controlled trials and implementation studies, including STEPSTONES [39, 47, 85, 92], have demonstrated the positive effects of structured, person-centered educational interventions on both clinical and psychosocial outcomes. However, the literature also highlights the need for greater standardization in delivering educational content and measuring outcomes across different phases of the transition process. Overall, this topic underscores the central role of education and individualized support as foundational elements in successful CHD transition programs.

#### Topic 2 – “Timing, Knowledge Transfer, and Developmental Needs”

Topic 2 captures a significant portion of the literature emphasizing the importance of initiating transitional care at the appropriate developmental stage and ensuring timely knowledge transfer to adolescents with CHD and their families (see detailed information from individual studies in Supplementary File 2). Studies within this topic conceptualize transition as a longitudinal process rather than a discrete event, where timing is critical for optimizing patient outcomes [6, 27, 30, 31, 33, 37, 38, 41–44, 58, 59, 62, 63, 77–80, 83, 87]. Several publications advocate for beginning transition discussions and interventions as early as ages 12 to 14, allowing sufficient time to align educational content with patients’ evolving cognitive, emotional, and social maturity [27, 37, 38, 83].

A core focus of this topic is knowledge transfer—not only about the disease itself but also about navigating adult care systems, understanding the importance of lifelong follow-up, and developing communication skills for more autonomous care management [43, 44]. Many studies highlight persistent knowledge gaps among adolescents nearing transfer and identify these as barriers to readiness and engagement in adult care [63]. Additionally, several contributions emphasize the role of developmentally appropriate education and health system navigation skills as essential prerequisites for a successful transition [30, 31, 33, 41, 48, 62, 87].

Some studies propose standardized quality indicators to guide the design and assessment of transition and transfer processes [6], while others recommend structured documentation, individualized planning, and targeted preparation for patients, caregivers, and healthcare providers [27]. However, the literature also points to wide variability in transition timing, institutional practices, and patient experiences, underscoring the need for harmonized, patient-centered protocols that accommodate developmental differences and ensure continuity of care.

This topic reinforces the understanding that effective transition care must be anticipatory, developmentally tailored, and grounded in knowledge empowerment, with adequate preparation time and system-level support to avoid care gaps and disengagement, completing what emerged in topic 1.

#### Topic 3 – “Transition Program Implementation and Coordination”

Topic 3 centers on the operational and organizational aspects of delivering structured transition programs for adolescents and young adults with CHD (see detailed information from individual studies in Supplementary File 2). This body of literature focuses on the design, coordination, and system-level integration of transition interventions, with particular attention to implementation logistics, role definition, and interdisciplinary collaboration [4, 5, 26, 29, 35, 36, 40, 54, 61, 64, 65, 67, 68, 70, 72, 75, 77, 83, 90].

Many of the included studies emphasize the role of dedicated transition coordinators, often nurses, who oversee the planning and delivery of transition activities and ensure continuity across pediatric and adult services [40, 64, 67, 77]. These programs are frequently multicomponent, involving structured planning sessions, individualized goal setting, peer support, and joint pediatric–adult clinic visits [26, 54]. Interventions are often informed by frameworks such as person-centered care, intervention mapping, or implementation science, and many are evaluated through randomized or quasi-experimental designs [4, 35, 75].

The literature on this topic also underscores the importance of implementation fidelity, adaptability, and contextual fit, which are critical factors for the sustainability and scalability of transition programs [4]. Despite growing consensus on core components, studies reveal significant variability in how programs are implemented, how responsibilities are distributed among team members, and how outcomes are tracked over time [4, 26]. System-level enablers, such as institutional commitment, cross-sector communication, and alignment with clinical guidelines, are frequently cited as prerequisites for successful implementation [4, 26, 70].

This topic reflects a shift from viewing transition as a primarily clinical or psychosocial task to recognizing it as a complex care coordination process requiring integration across disciplines, settings, and care models. It highlights the need for structured, reproducible transition frameworks that are supported by robust evaluation strategies and embedded within broader health system infrastructures.

#### Topic Validation Using MCA

**Fig. 3:**
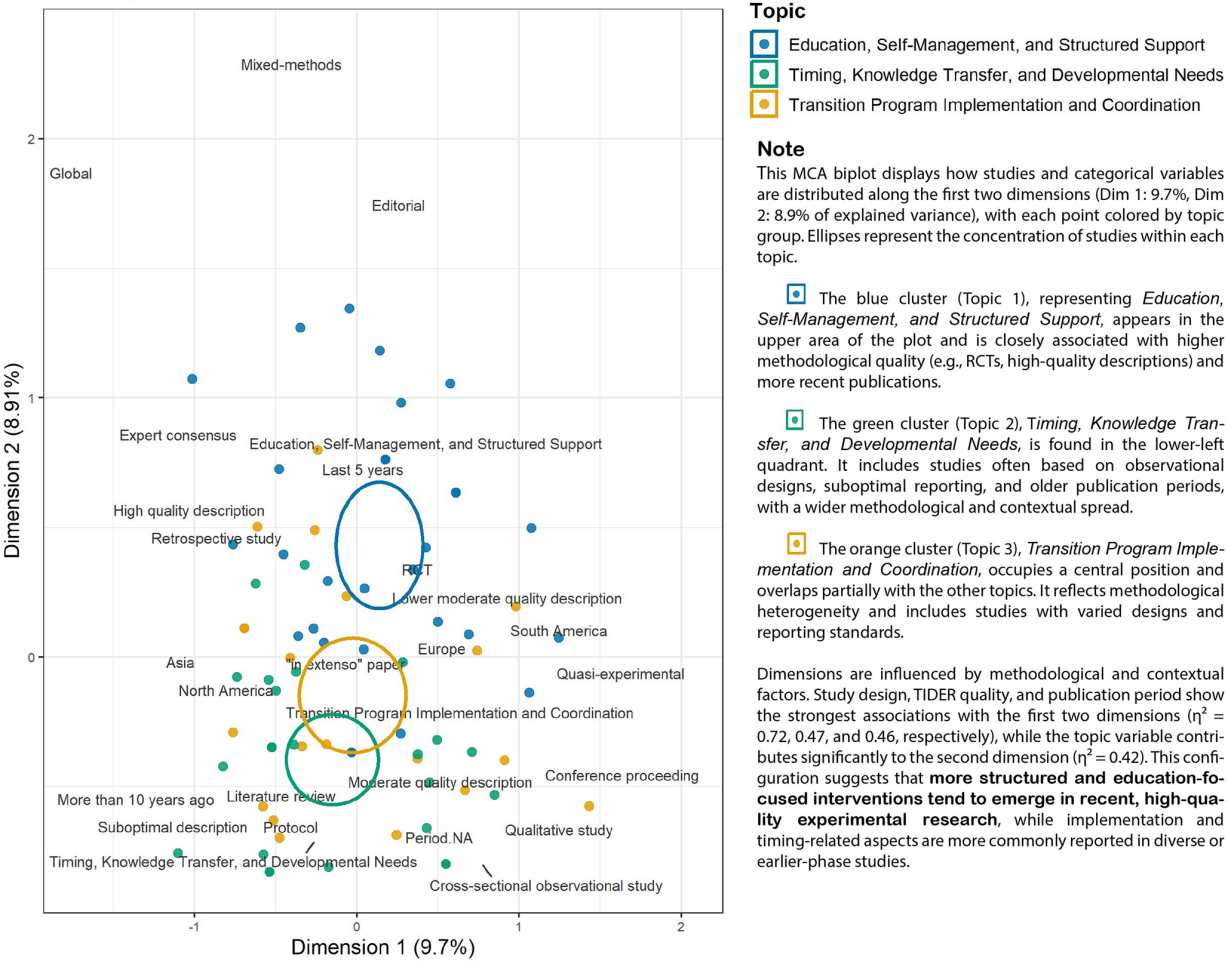
Multiple correspondence analysis biplot of topics and variables to determine trends. Note: This MCA biplot illustrates the relationships between included articles and variables (topic, publication period, categorized TIDieR score, continent, and study type). Each point represents a study, colored by topic, and ellipses represent the concentration (95% confidence interval) of studies within each topic group. Dimensions 1 and 2 explain 9.7% and 8.91% of the variance, respectively. Results show topic-specific associations with methodological quality, study type, and temporal distribution. Topic 1 clusters with recent and high-quality studies, while Topic 2 is linked to broader methodological variation

MCA allowed us to visualize the spatial relationships between the included literature with topics, countries (categorized as the continent of origin), quality of description of the intervention (TIDieR completeness score), and publication period (Fig. 3).

Figure 3 displays the biplot from MCA, mapping how studies and categorical variables are distributed along the first two dimensions (Dimension 1 = 9.7%, Dimension 2 = 8.9% of explained variance). Each dot represents a study and is color-coded by thematic topic, while labeled categories indicate associated methodological or contextual features. Ellipses visually group studies by topic, showing their relative proximity and internal variability.

The blue cluster (Topic 1), Education, Self-Management, and Structured Support, is concentrated in the upper region and is closely associated with recent publications and higher methodological quality (i.e., randomized controlled trials with high-quality intervention descriptions).

The green cluster (Topic 2), Timing, Knowledge Transfer, and Developmental Needs, appears in the lower-left quadrant and includes studies with suboptimal reporting, older publication dates, and a broader contextual spread.

The orange cluster (Topic 3), Transition Program Implementation and Coordination, occupies a more central position and overlaps with the other clusters. It reflects methodological heterogeneity and includes a range of study types and reporting standards.

The plot reveals that study design (η² = 0.72, *p* < .001), quality of the description of intervention (TIDieR; η² = 0.47, *p* < .001), and publication period categorized in three ordinal categories (i.e., “Last 5 years”, “6–10 years ago”, “More than 10 years ago”; η² = 0.46, *p* < .001) are the strongest contributors to the first two dimensions. The topic variable contributes significantly to Dimension 2 (η² = 0.42, *p* < .001). These findings suggest that structured, education-focused interventions are more likely to emerge in recent, high-quality experimental research, while implementation and timing-related interventions are more frequently reported in diverse or earlier-phase studies.

## Discussion

This scoping review systematically maps the current literature, highlighting innovative areas and emerging trends while also identifying critical gaps, particularly in clinical, psychosocial, and organizational aspects. We identified 73 articles that described a comprehensive overview of key trends in transitional care literature for pediatric and young patients with CHD. Through systematic analysis, we identified three core themes: (1) Education, Self-Management, and Structured Support; (2) Timing, Knowledge Transfer, and Developmental Needs; and (3) Transition Program Implementation and Coordination. The main contribution of this study lies in its integration of data mining techniques, such as LDA and MCA, uniquely applied to map a large body of literature systematically. This approach enables the identification of nuanced and emerging topics and their trends within CHD transitional care.

The topic *Education*,* Self-Management*,* and Structured Support* highlights the importance of preparing adolescents with CHD to assume a proactive role in managing their condition [2, 9, 39, 45, 47, 49, 50, 52, 53, 55–57, 60, 66, 71, 73, 74, 76, 81, 82, 84–86, 88, 89, 91–93]. This literature emphasizes patient education as a core mechanism to enhance self-efficacy, health literacy, and long-term adherence. Interventions often involve structured curricula, transition readiness assessments, digital tools, and counseling sessions aimed at improving disease knowledge and autonomy. Studies related to this topic suggest that education and self-management support are essential for transition readiness and contribute to improved health outcomes and reduced care discontinuity. Family involvement and psychosocial support are often integrated into these interventions, reflecting the multifaceted nature of adolescent development and the challenges of navigating a chronic condition into adulthood. However, findings also suggest inconsistencies in how these elements are operationalized, and there is a need for greater standardization of educational content, delivery formats, and measurement of long-term outcomes [94, 95].

The topic *Timing*,* Knowledge Transfer*,* and Developmental Needs* captures a conceptual shift from transition as a fixed event to a flexible, developmentally appropriate process [6, 27, 30, 31, 33, 37, 38, 41–44, 58, 59, 62, 63, 77–80, 83, 87]. This topic emphasizes the need to initiate transition planning early, often in early adolescence, and to tailor interventions to individual cognitive, emotional, and social maturity. Many studies focus on readiness assessment, knowledge gaps, and perceived preparedness among both patients and families [4, 6, 36, 40]. Others propose system-level solutions, such as the development of quality indicators, coordinated referral systems, and standardized documentation practices [75]. The literature also highlights that delayed or unstructured transitions are associated with care disruptions and decreased follow-up rates [61]. Evidence from this theme calls for greater attention to the timing and pacing of transition interventions and points to the value of longitudinal engagement across adolescent development. A strong focus on information dissemination reflects an emerging priority in the field, likely in response to the recognized challenges that young patients with CHD face in managing their health independently. These findings highlight how the field of transitional care for CHD is evolving to encompass more comprehensive, multi-faceted approaches, addressing both individual and family needs while prioritizing education as a key element for successful transition outcomes. Notably, this theme includes meta-analyses and policy-focused publications that advocate for integrated frameworks and strategic implementation [27, 28, 30, 31, 41, 42].

The topic of *Transition Program Implementation and Coordination* encompasses a substantial body of evidence on the design, delivery, and oversight of structured transition programs [4, 5, 26, 29, 35, 36, 40, 54, 61, 64, 65, 67, 68, 70, 72, 75, 77, 83, 90]. These studies often focus on multicomponent interventions delivered through standardized protocols and supported by dedicated professionals, such as transition coordinators. Emphasis is placed on ensuring continuity between pediatric and adult services, often through the use of individualized transition plans, process evaluations, and implementation fidelity assessments. This literature reflects growing international consensus around the value of structured transition pathways, particularly when underpinned by person-centered care principles and supported by interdisciplinary collaboration. Programs like STEPSTONES exemplify this approach [49, 85, 91], demonstrating improvements in empowerment, knowledge, and follow-up continuity through randomized controlled trials within hybrid experimental designs [54, 91]. Despite these advancements, gaps remain in terms of real-world scalability and integration of such programs into routine care across diverse health systems.

While the current body of research predominantly focuses on disease management and psychosocial aspects in isolation, integrated care could unify clinical, psychosocial, and educational interventions, ensuring a more seamless and patient-centered transition [96]. However, evidence of fully realized integrated care models in CHD transitional care remains limited, underscoring the need for future research and policy efforts to develop and evaluate such approaches [40]. In this context, integrated care has the capacity to address the unique needs of patients with CHD and their families comprehensively, improving both health outcomes and the overall transition experience [96].

Taken together, these three topics reflect a field that is progressively evolving toward more comprehensive and person-centered approaches to transitional care, considering the achievement of a solid base of evidence (Class 1, Level A) recommending transitional care in young individuals with CHD [50]. The three topics also point to areas where integration remains limited. While implementation and education are often studied in isolation, combining these with considerations of developmental timing and knowledge transfer could create more holistic transition models. Our MCA analysis reinforces this, showing how methodological features, such as study design and quality of intervention reporting, correlate strongly with thematic clusters.

The lexicometric properties of the corpus further illustrate the field’s conceptual richness and complexity [97]. The high type-token ratio (26%) and the large number of hapax legomena point to a wide range of specialized vocabulary and distinctive research approaches across studies. This linguistic diversity aligns with the interdisciplinary nature of transitional care, encompassing cardiology, nursing, psychology, and health policy domains [98].

Our findings underscore critical gaps in the detailed reporting of transition interventions, particularly in terms of intervention fidelity and tailoring to individual needs. Lower completeness rates in the TIDieR checklist items indicate that many studies lack comprehensive descriptions of essential intervention components, which limits the replicability of transition programs and complicates the assessment of their effectiveness. Detailed reporting on each element—such as materials used, frequency, duration, and adaptation processes—is necessary to enhance transparency and to establish best practices [15]. In addition, information on how interventions were tailored to meet participants’ specific needs is frequently missing, as is documentation of any modifications made during the study, including the rationale and methods for these changes. This lack of detail impedes understanding of how interventions may need to be adapted in practice. Additionally, the reporting of intervention fidelity in terms of planned and actual adherence is often overlooked. Fidelity studies, which assess whether interventions are delivered as intended and outline strategies to maintain adherence, are essential for determining an intervention’s reliability and scalability. Addressing these reporting gaps would strengthen the evidence base and support the development of individualized, effective transition programs for CHD patients.

## Implications for Practice and Research

The findings of this study highlight the importance of implementing structured, evidence-based transition programs tailored to the unique needs of adolescents with CHD and their families. Healthcare providers are able to better support young patients through the complexities of transitioning to adult care by developing adaptable transition models. Improved documentation practices, aligned with the TIDieR framework, are crucial for enhancing the reproducibility and fidelity of these interventions. Such practices would allow for a more accurate assessment of program effectiveness and facilitate the dissemination of best practices across institutions, leading to more consistent and high-quality transitional care. Additionally, this study lays the groundwork for future outcome-based research that could further evaluate the effectiveness of specific transitional care models.

Future studies could focus on longitudinal assessments of patient-reported outcomes, including health literacy, empowerment, self-management skills, and quality of life metrics, to better understand the long-term impact of transition programs. Moreover, research could explore the role of psychosocial support interventions, parental involvement, and the integration of digital health tools, such as mobile apps or telemedicine, in enhancing the transition process. A significant step forward would be the development of a core outcome set (COS) for transitional care, ensuring consistent measurement and reporting across studies. A COS would help establish standardized outcomes that are meaningful to patients, caregivers, and healthcare providers, facilitating comparisons between interventions and supporting the synthesis of evidence across diverse contexts. This would greatly enhance the ability to evaluate and refine transition programs, paving the way for more robust, evidence-based practices in this critical area of care. Investigating cross-institutional collaborations and standardized protocols for transitional care delivery could further support the development of scalable, adaptable models applicable across diverse healthcare systems. Finally, there is a need for research that addresses disparities in access to transitional care, ensuring that interventions are equitable and culturally sensitive to meet the needs of diverse patient populations.

The current study has several limitations. First, as a scoping review, it does not assess the quality of the included studies, which limits the ability to draw firm conclusions about the effectiveness of specific transition interventions. Additionally, while ensuring comprehensive coverage, the broad inclusion criteria may introduce variability in study design, outcomes, and reporting standards, making comparing findings directly across studies challenging. The reliance on existing literature also means that emerging or unpublished models of transitional care may not be represented. Additionally, while topic modeling provided valuable insights into thematic trends, the approach is dependent on the quality and consistency of reporting in the original studies, potentially limiting the depth of analysis on nuanced aspects of each intervention. These limitations underscore the need for future systematic reviews on outcomes and quality assessment of transition programs for patients with CHD.

Another important limitation concerns the limited attention paid in the existing literature to the clinical and developmental heterogeneity of individuals with CHD. CHD encompasses a wide spectrum of anatomical complexity, comorbidities, and neurodevelopmental conditions, including genetic syndromes and acquired neurological impairments, which may substantially influence transition readiness and the feasibility of independent disease management. Across the included studies, patient-level clinical characteristics were inconsistently reported, and few investigations explicitly addressed how lesion severity, cognitive impairment, or special healthcare needs were incorporated into transition models or analyses. In several prospective and intervention-based studies, individuals with significant developmental delay or complex psychosocial needs were either excluded or insufficiently characterized, limiting the generalizability of reported transition outcomes. This gap highlights a critical shortcoming of the current evidence base and underscores the need for future research to explicitly account for patient complexity and vulnerability in the design, implementation, and evaluation of transitional care programs for CHD.

## Conclusion

This scoping review offers a comprehensive synthesis of the literature on transitional care for adolescents and young adults with CHD, identifying three core thematic areas from 73 included records. These findings highlight the growing emphasis on structured, theory-informed transition models, the critical role of patient and family education, and the importance of developmentally appropriate, individualized care. At the same time, the review reveals somewhat persistent gaps, particularly in the standardization of outcome measures and the fidelity of intervention reporting, that limit the scalability and reproducibility of transition programs.

Future research should prioritize the development of a core outcome set to harmonize the evaluation of transition interventions, along with enhanced reporting practices to improve intervention fidelity and replicability. Longitudinal studies are needed to assess the sustained impact of transitional care across the life course. In parallel, policy initiatives should focus on ensuring equitable access to transition services, fostering cross-institutional and international collaboration, and supporting the implementation of flexible, culturally responsive models that address the diverse needs of populations with CHD and their families. 

## Key References

- Moons P, Bratt E-L, De Backer J, et al. Transition to adulthood and transfer to adult care of adolescents with congenital heart disease: a global consensus statement. Eur Heart J. 2021;42:4213–4223.
  - ○ This global consensus provides a comprehensive framework for transition in CHD, highlighting the need for structured, standardized programs and international collaboration.
- Thomet C, Schwerzmann M, Budts W, et al. Transfer and transition practices in 96 European adult congenital heart disease centres. Int J Cardiol. 2021;328:89–95.
  - ○ This multicenter survey maps current practices across Europe, revealing heterogeneity and critical gaps in the organization of transition care.
- Saarijärvi M, Wallin L, Moons P, Gyllensten H, Bratt E-L. Mechanisms of impact and experiences of a person-centred transition programme for adolescents with CHD: the Stepstones project. BMC Health Serv Res. 2021;21:573.
  - ○ This study evaluates a structured, person-centered transition program, providing evidence of its impact on self-management and empowerment among adolescents with CHD.

## Supplementary Information

Below is the link to the electronic supplementary material.

Supplementary File 1 (DOCX 24.7 KB)

Supplementary File 2 (DOCX 67.3 KB)

Supplementary File 3 (DOCX 58.3 KB)

## Data Availability

No datasets were generated or analysed during the current study.
