# Supplementary material for: Bridging Pediatric to Adult Care: A Scoping Review on Transitional Care for Individuals with Congenital Heart Disease Using Data Mining Techniques to Identify Key Topics
Source: Curr Cardiol Rep. 2026 May 22;28(1):57. doi: 10.1007/s11886-026-02377-1 (PMC13194312; doi:10.1007/s11886-026-02377-1)
Supplement: Supplementary file 1 — (DOCX 24.7 KB) [file 11886_2026_2377_MOESM1_ESM.docx]

**Supplementary File 1**. Complete search strategy

Database: Medline® (accessed via PubMed)

| ("heart defects, congenital"[MeSH Terms] OR "Heart Diseases/congenital"[Mesh] OR ((heart*[tiab] OR cardiac[tiab]) AND (defect*[tiab] OR disease*[tiab] OR malformat*[tiab] OR abnorm*[tiab] OR pathol*[tiab]) AND congenital[tiab])OR "congenital heart defect*"[tiab] OR "congenital heart disease*"[tiab] OR chd) AND ("transitional care"[MeSH Terms] OR transit*[tiab] OR "Transition to Adult Care"[Mesh] OR "transition to adult care"[tiab] OR transfer*[tiab]) AND (adolescence OR young OR youngster OR youth OR teen* OR girl* OR boy OR boys OR pediatr* OR minor* OR juvenile) |
| --- |

Clean results: 1745 (28/02/2025)

SEARCH DETAILS:

("heart defects, congenital"[MeSH Terms] OR "heart diseases/congenital"[MeSH Terms] OR (("heart*"[Title/Abstract] OR "cardiac"[Title/Abstract]) AND ("defect*"[Title/Abstract] OR "disease*"[Title/Abstract] OR "malformat*"[Title/Abstract] OR "abnorm*"[Title/Abstract] OR "pathol*"[Title/Abstract]) AND "congenital"[Title/Abstract]) OR "congenital heart defect*"[Title/Abstract] OR "congenital heart disease*"[Title/Abstract] OR ("cancer health disparities"[Journal] OR "chd"[All Fields])) AND ("transitional care"[MeSH Terms] OR "transit*"[Title/Abstract] OR "Transition to Adult Care"[MeSH Terms] OR "Transition to Adult Care"[Title/Abstract] OR "transfer*"[Title/Abstract]) AND ("adolescences"[All Fields] OR "adolescency"[All Fields] OR "adolescent"[MeSH Terms] OR "adolescent"[All Fields] OR "adolescence"[All Fields] OR "adolescents"[All Fields] OR "adolescent s"[All Fields] OR ("young"[All Fields] OR "youngs"[All Fields]) OR ("youngster"[All Fields] OR "youngster s"[All Fields] OR "youngsters"[All Fields]) OR ("adolescent"[MeSH Terms] OR "adolescent"[All Fields] OR "youth"[All Fields] OR "youths"[All Fields] OR "youth s"[All Fields]) OR "teen*"[All Fields] OR "girl*"[All Fields] OR ("men"[MeSH Terms] OR "men"[All Fields] OR "boy"[All Fields]) OR ("men"[MeSH Terms] OR "men"[All Fields] OR "boys"[All Fields]) OR "pediatr*"[All Fields] OR "minor*"[All Fields] OR ("juvenile"[All Fields] OR "juvenile s"[All Fields] OR "juveniles"[All Fields] OR "juvenility"[All Fields]))

Database: Embase (accessed via Elsevier)

| ('congenital heart malformation'/exp OR 'congenital heart malformation' OR 'heart disease'/exp/dm_cn OR 'heart disease' OR (('heart*':ti,ab OR 'cardiac':ti,ab) AND ('defect*':ti,ab OR 'disease*':ti,ab OR 'malformat*':ti,ab OR 'abnorm*':ti,ab OR 'pathol*':ti,ab) AND 'congenital':ti,ab) OR 'congenital heart defect*':ti,ab OR 'congenital heart disease*':ti,ab OR 'chd') AND ('transitional care'/exp OR 'transitional care':ti,ab OR 'transit*':ti,ab OR 'transition to adult care'/exp OR 'transition to adult care':ti,ab OR 'transfer*':ti,ab) AND ('adolescence'/exp OR adolescen*:ti,ab OR young*:ti,ab OR 'youth'/exp OR 'youth':ti,ab OR 'teen*':ti,ab OR 'girl*':ti,ab OR 'boy'/exp OR boy*:ti,ab OR 'pediatr*':ti,ab OR 'minor*':ti,ab OR 'juvenile'/exp OR 'juvenile':ti,ab) AND ([article]/lim OR [article in press]/lim OR [conference paper]/lim OR [conference review]/lim OR [review]/lim) |
| --- |

Clean results: 2716 (28/02/2025)

Database: CINAHL Complete (accessed via EBSCOhost)

| TI ( (((heart* OR cardiac) AND (defect* OR disease* OR malformat* OR abnorm* OR pathol*) AND congenital) OR "congenital heart defect" OR “congenital heart defects” OR "congenital heart disease" OR "congenital heart diseases" OR chd) ) OR AB ( (((heart* OR cardiac) AND (defect* OR disease* OR malformat* OR abnorm* OR pathol*) AND congenital) OR "congenital heart defect" OR “congenital heart defects” OR "congenital heart disease" OR "congenital heart diseases" OR chd) ) OR (MH "Heart Defects, Congenital") AND TI ( transit* OR "transition to adult care" OR transfer* ) OR AB ( transit* OR "transition to adult care" OR transfer* ) OR (MH "Transitional Care") AND TI ( adolescen* OR young* OR youth OR teen* OR girl* OR boy* OR pediatr* OR minor* OR juvenil* ) OR AB ( adolescen* OR young* OR youth OR teen* OR girl* OR boy* OR pediatr* OR minor* OR juvenil* ) OR (MH "Adolescence") |
| --- |

Filters/Limiters/Expanders:

- Expanders - Apply equivalent subjectsEnglish
- Search modes - Boolean/Phrase

Filtered results: **263** (28/02/2025)

Database: PsycINFO

| TI ( (((heart* OR cardiac) AND (defect* OR disease* OR malformat* OR abnorm* OR pathol*) AND congenital) OR "congenital heart defect" OR “congenital heart defects” OR "congenital heart disease" OR "congenital heart diseases" OR chd) ) OR AB ( (((heart* OR cardiac) AND (defect* OR disease* OR malformat* OR abnorm* OR pathol*) AND congenital) OR "congenital heart defect" OR “congenital heart defects” OR "congenital heart disease" OR "congenital heart diseases" OR chd) ) OR DE "Heart Disorders" AND TI ( transit* OR "transition to adult care" OR transfer* ) OR AB ( transit* OR "transition to adult care" OR transfer* ) OR TI ( adolescen* OR young* OR youth OR teen* OR girl* OR boy* OR pediatr* OR minor* OR juvenil* ) OR AB ( adolescen* OR young* OR youth OR teen* OR girl* OR boy* OR pediatr* OR minor* OR juvenil* ) |
| --- |

Filters/Limiters/Expanders:

- Expanders - Apply equivalent subjectsEnglish
- Search modes - Boolean/Phrase

Filtered results: 65 (28/02/2025)

Database: Cochrane review

| [Heart Defects, Congenital] OR [Heart Diseases] OR (((heart* OR cardiac) AND (defect* OR disease* OR malformat* OR abnorm* OR pathol*) AND congenital) OR chd) AND [Transitional Care] OR [Transition to Adult Care] OR (transit* OR transfer* OR "Transition to Adult Care") AND [Adolescent]OR (adolescen* OR young* OR youth* OR teen* OR girl* OR boy* OR pediatr* OR minor* OR juvenil*) |
| --- |

Filters:

- ti,ab,kw
- explode all trees

Filtered results: **96** (28/02/2025)
