# Supplementary material for: Bridging Pediatric to Adult Care: A Scoping Review on Transitional Care for Individuals with Congenital Heart Disease Using Data Mining Techniques to Identify Key Topics
Source: Curr Cardiol Rep. 2026 May 22;28(1):57. doi: 10.1007/s11886-026-02377-1 (PMC13194312; doi:10.1007/s11886-026-02377-1)
Supplement: Supplementary file 2 — (DOCX 67.3 KB) [file 11886_2026_2377_MOESM2_ESM.docx]

| Supplementary File 2. Summary of the included studies | | | | | | | |
| --- | --- | --- | --- | --- | --- | --- | --- |
| Authors and year \| types of publications | **Country** | **Main objective** | **Study design** | **Sample** | **Transition characteristics** | **Key findings** |  |
| Bratt et al. (2023)  Journal Article | Sweden | To assess the empowering effect of a structured, person-centered transition program for adolescents with congenital heart disease (CHD) and examine its impact on transition readiness, health outcomes, and parental involvement. | Hybrid experimental design: randomized controlled trial (RCT) within a longitudinal observational study. | 208 patients, ages 16 to 18.5; divided into IG (70), CG (69), CCCG (69), with dropout rates: IG (16), CG (8), CCCG (29). The majority had complex or moderate CHD. | Transitional care program in outpatient pediatric cardiology, with outcome assessments at T0 (16 years), T1 (17 years), and T2 (18.5 years). Administered by trained, specialized nurses. | Empowerment increased in IG compared to CG (Δ 3.44). IG observed improved parental involvement, CHD knowledge, and physical appearance satisfaction. No contamination detected between CG and CCCG. |  |
| Acuña Mora et al. (2022)  Journal Article | Sweden | To examine the directional effects between patient empowerment and patient-reported outcomes (PROs), including communication skills, health, quality of life, and transition readiness in adolescents with CHD. | RCT embedded within a longitudinal observational study (RI-CLPM model). | 140 patients, ages 16 to 18.5, with CHD severity: mild (13.8%), moderate (64.6%), complex (24.6%). IG and CG with 70 each; significant dropouts at each follow-up. | Three time-point interventions in outpatient pediatric cardiology, assessing empowerment, communication skills, health, and readiness for transition. | Empowerment, communication skills, patient health, and transition readiness improved significantly, particularly from T1 (17 years) to T2 (18.5 years), showing that readiness for transition correlates with age. A significant link was observed between empowerment and communication skills; empowerment is also proportional to transition readiness. |  |
| Bratt et al. (2022)  Conference proceeding | Sweden | To investigate the empowering effect of a structured transition program on adolescents with CHD and assess its impact on secondary outcomes like parental involvement, specific CHD knowledge, and satisfaction with physical appearance. | Hybrid experimental design: RCT embedded in a longitudinal observational study across seven CHD centers in Sweden. | 138 adolescents with CHD, aged 16 at baseline up to 18.5 in the follow-ups, who participated along with their parents. This group was divided across randomized and observational study arms for assessments over time. | Three time-point interventions (T0 at 16 years, T1 at 17 years, and T2 at 18.5 years) were conducted in a hospital setting, focusing on transition empowerment and parental involvement. | Significant improvement in empowerment in IG from T0 to T2. Secondary outcomes, including parental involvement, CHD knowledge, and satisfaction with appearance, also showed significant positive change, supporting the program's effectiveness for patients and caregivers. |  |
| Ladouceur et al. (2016)  Conference proceeding | France | To evaluate the effectiveness of a structured educational program on knowledge and self-management skills among adolescents and young adults with CHD, focusing on transition preparation and quality of life improvement. | A monocentric, quasi-experimental design with quantitative and qualitative elements. | 115 adolescents and young adults with CHD (45 girls); IG: 22 in an educational program for 11 months; CG: 93 in usual care. | A specific knowledge questionnaire assessed educational intervention to improve knowledge and self-management. Conducted in a hospital setting. | IG showed a higher mean knowledge score (11.7) than CG (8.6). CG had significant knowledge gaps, with only 20% aware of follow-up guidelines and 43% aware of infection prevention. The program effectively improved follow-up and cardiac symptoms knowledge, enhancing quality of life. |  |
| Charles et al. (2016)  Conference proceeding | Canada | To evaluate the impact of a structured, nurse-led intervention on self-management and transition readiness in adolescents with moderate to complex CHD, emphasizing tools like MyHealth Passport and skill development. | Multicentric RCT with embedded qualitative elements. | 57 adolescents, ages 16-17, with moderate to complex CHD. | Two-session intervention: Session 1 (1 hour) focused on patient education, MyHealth Passport creation, and goal setting; Session 2 (1.5 hours, two months later) emphasized self-management skills through videos, role-play, and goal follow-up. Conducted in hospital. | Key steps: MyHealth Passport creation, goal setting, and role-play. 78% kept their passport, 46% felt ready for transition post-intervention, versus 5% initially. 40% requested additional sessions to further understand and manage their condition, highlighting the need to begin transition preparation early. |  |
| Mondal et al. (2020)  Journal Article | Canada | To determine the successful transfer rate of adolescents with CHD from pediatric to adult care and identify patient and context-related factors associated with unsuccessful transfer. | Retrospective cohort study at a tertiary care center. | 279 patients, ages 17-26, with CHD severity: mild (48%), moderate (38%), and severe (14%). | Use transition pamphlets, MyTransition mobile app with Transition-Q questionnaire, and nurse-led follow-up with reminders via mail and phone to ensure attendance at adult care. | 96.4% successfully transferred, with only 3.6% lost to follow-up. Risk factors for loss: male sex, >200 km distance to clinic, and mild CHD severity. Early discussions and transition tools, like the MyTransition app, were associated with high transfer success. |  |
| Sanker et al. (2021)  Conference proceeding | United States | To assess self-preparedness for the transition to adult care among adolescents and young adults with CHD, evaluating their confidence and knowledge about health management. | Cross-sectional, survey-based study at a single cardiology center. | 88 respondents out of 396 eligible patients, ages 12-25, with CHD. | A transition readiness survey (AAP/ACP Transition Readiness for Youth Assessment) was mailed to patients to assess their confidence and knowledge of health management. Conducted at a pediatric heart center. | 85% felt confident in managing their health, but 15% lacked an understanding of lifelong care, 14% didn’t grasp the need for health insurance, and 54% required assistance with medication refills. Gaps in younger patients highlight a need for targeted health education to enhance transition readiness. |  |
| Mackie et al. (2018)  Journal Article | Canada | To evaluate the impact of a nurse-led transition intervention on reducing delays in adult CHD care and improving CHD knowledge, self-management, and self-advocacy skills in adolescents. | Monocentric, RCT | 121 adolescents, ages 16-17, with moderate or complex CHD (58 in IG, 63 in CG). | Two individualized sessions for the IG: 1st session (15 min) on MyHealth Passport and CHD education; 2nd session (11 min) on self-management skills; follow-up assessments at 1, 6, 12, and 18 months. Conducted in a pediatric cardiology clinic. | IG had shorter wait times for adult care (2 months vs. 7 in CG) and higher scores on MyHeart knowledge and TRAQ self-management and self-advocacy measures. 94% of IG answered questions independently vs. 67% in CG, indicating improved preparation. Younger patients showed knowledge gaps, emphasizing the need for early transition education. |  |
| Acuña Mora et al. (2020)  Journal Article | Sweden​ | To empower adolescents with CHD and facilitate their transition to adult care through a person-centered program developed via Intervention Mapping. | Hybrid experimental design (RCT embedded in a longitudinal study). | Young persons with CHD, ages 14-25, and their parents. | Three meetings over 2.5 years with a transition coordinator. The program uses goal-setting, modeling, and active learning to improve knowledge, self-efficacy, and self-management. | The program aims to increase patient empowerment, prepare adolescents to take responsibility for their health , and enhance knowledge, self-efficacy, and self-management. Evaluation of effectiveness and process is ongoing. |  |
| Charles et al. (2021)  Journal Article | Canada | To explore the effectiveness of a two-session nurse-led intervention on transition readiness among adolescents with CHD, focusing on key elements such as health passport creation, goal setting, and role-play exercises. | Mixed-methods, two-site clinical trial. | 57 adolescents, ages 16-17, with moderate or complex CHD. | Two 1-on-1 sessions: Session 1 focused on education and creating a health passport; Session 2 emphasized self-management, goal setting, and role-plays. Conducted by trained RNs at two pediatric cardiology centers. | Identified a typology of transition readiness: 1) Independent, 2) Ready, 3) Follow-up Needed, and 4) At-risk. About 40% needed additional transition support. Key components valued were the MyHealth Passport, goal setting, and role-plays. |  |
| Burström et al. (2019)  Journal Article | Sweden | To describe the level of transition readiness in adolescents with CHD, compare adolescents’ assessments with their parents, and study factors influencing transition readiness. | Cross-sectional, triadic evaluation. | 157 adolescents with CHD, ages 14-18, and their parents (complete triads: adolescent, mother, and father). | Transition Readiness Questionnaire (RTQ) assessed adolescent responsibility, parental involvement, and overall readiness for transfer. | Adolescents rated themselves as more ready than their parents rated them. Higher readiness correlated with older age, higher empowerment, and less perceived threat from the illness. Parental involvement decreased with age. Enhanced transition readiness is linked with parental trust and lower perceived illness threat. |  |
| Campbell et al. (2016)  Journal Article | UK, Canada, Australia, USA | To evaluate the effectiveness of interventions designed to improve the transition of care for adolescents from pediatric to adult health services. | Systematic review of 4 RCTs (N=238) | Adolescents with various chronic conditions (heart disease, spina bifida, type 1 diabetes, cystic fibrosis, IBD); 4 RCTs included from USA, Canada, and Australia. | Interventions included nurse-led sessions, a web/SMS program, structured phone support post-transfer, and a two-day workshop. Most focused on education, self-management, and transition readiness. | Low-certainty evidence: slight improvements in disease knowledge, self-efficacy (PAM), and TRAQ scores with nurse-led and tech-based interventions. No significant improvements in health outcomes, QoL, or transfer rates. Highlights need for more rigorous, long-term studies across diverse conditions and systems. |  |
| Flocco et al. (2019)  Journal Article | Italy | To assess the impact of a transition care model on health perceptions in adolescents with CHD, focusing on quality of life, satisfaction, knowledge, and anxiety reduction. | Quasi-experimental, pre/post-intervention study | 224 adolescents with CHD (60.7% male; mean age: 14.84 years), classified as simple (22%), moderate (56%), or severe (22%) CHD. | A three-pillar transition model: education on CHD, counseling sessions with peer support, and coordinated care led by a transition coordinator. Conducted at an Italian center for CHD. | Significant improvements in pain reduction, anxiety, knowledge, life satisfaction, and quality of life (all p < 0.001). The analyses showed promise in enhancing health perceptions for adolescent CHD patients. |  |
| Fernandes et al. (2019)  Journal Article | United States | To determine adolescents’ and young adults’ understanding of the need for lifelong cardiac care (LLCC) and awareness of recommended adult care providers for CHD. | Multi-center, cross-sectional survey study | 290 adolescents and young adults, ages 13-20, with surgically repaired CHD (62% male). Diagnoses include repaired aortic coarctation, tetralogy of Fallot, Fontan procedure, and arterial switch operation for transposition of the great arteries. | A survey was administered at 10 U.S. medical centers with ACHD programs, assessing knowledge of LLCC and provider types for adult care. | 78% understood the need for LLCC, but only 37% knew an ACHD specialist should guide it. Only 37% recalled discussions about LLCC with their care team, but 90% desired more information. Indicates a need for targeted LLCC education. |  |
| Gaydos et al. (2020)  Journal Article | United States | To evaluate the impact of a transition clinic intervention on follow-up rates in adolescents and young adults with CHD, aiming to reduce the rate of patients lost to follow-up. | Retrospective case-control study within a quality improvement project | 53 adolescents and young adults with CHD, aged ≥11 years, from a single-center CHD clinic | Monthly transition clinic with self-assessment questionnaires, focused teaching, follow-up via a clinic registry, and personalized ACHD referrals. | The "lost to follow-up" rate in the intervention group was significantly lower (7.3%) compared to the control (25.9%, *p* < 0.01). Transition readiness increased with age, and clinic participation was the primary factor associated with improved follow-up. |  |
| Ladouceur et al. (2017)  Journal Article | France | To evaluate the educational needs and impact of a structured transition intervention program on knowledge and self-management skills among adolescents with CHD. | Cross-sectional study with education vs. control group | 115 adolescents with CHD (mean age 17 ± 2 years; 47 females) enrolled at a pediatric cardiology clinic in Paris | Structured education program with sessions on health issues, risky behaviors, self-management, and group sessions on common topics. Materials included a "Health Passport" and video game training. | The education group scored significantly higher on knowledge (11.7 ± 3.5) than the control (8.6 ± 3.2, p < 0.001). Key gaps in the control group included knowledge of the condition, follow-up needs, and pregnancy risks. |  |
| Mackie et al. (2014)  Journal Article | Canada | To assess the impact of a nurse-led transition intervention on self-management and knowledge about heart disease among adolescents with CHD. | Clinical trial | 58 adolescents with moderate or complex CHD or cardiomyopathy, ages 15-17 | The 1-hour individualized, nurse-led session focused on CHD education, creation of MyHealth Passport, and transition skills training. | The intervention group had significantly higher self-management scores on the TRAQ and improved MyHeart knowledge scores (75% vs. 61% in controls) at the 6-month follow-up, showing enhanced readiness for transition. |  |
| Soto et al. (2020)  Journal Article | Chile | To evaluate transition readiness and factors impacting the successful transfer from pediatric to adult care in adolescents and young adults with CHD. | Multi-center, observational study | Adolescents and young adults with CHD across various severities | Comprehensive transition readiness assessment using structured surveys, self-management evaluations, and timely follow-up tracking. | Findings indicated a positive correlation between structured transition preparation and increased follow-up attendance. Specific factors such as parental involvement, understanding of health management, and healthcare provider communication significantly improved transition outcomes. |  |
| Ricci et al. (2023)  Journal Article | United Kingdom | To evaluate the impact of a nurse-led transition service on knowledge and self-care skills in adolescents and young adults with CHD and identify factors associated with a successful transition to adult care. | Retrospective longitudinal study | 592 adolescents and young adults with CHD, mean age 15.2 years, including patients with learning and physical disabilities | Nurse-led clinics using a structured model with 2-3 clinic visits over several years, addressing CHD knowledge, self-care, and health independence | Patients without learning disabilities showed significant improvement in knowledge (e.g., naming/understanding their CHD) from first to third visits. Successful transition is associated with younger age, simpler CHD, and the absence of physical disability. |  |
| Flocco et al. (2018)  Journal Article | Italy | To evaluate the effects of a standardized educational intervention on health perception, quality of life, and satisfaction in adolescents with CHD transitioning to adult care. | Quasi-experimental, pre-post design | 100 adolescents with CHD, ages 14-21 (mean age 14.79 years, 60% male) | The multidisciplinary transition clinic model has three phases: clinical condition education, psychological support, peer counseling, and communication enhancement through a Transition Coordinator. | Significant improvements at follow-up in pain/discomfort, anxiety/depression, health perception, general satisfaction, and quality of life. Results indicate that a structured, CHD-specific transition program benefits adolescent health outcomes. |  |
| de Hosson et al. (2024)  Journal Article | Belgium | To evaluate the effectiveness of the TWAH (Transition With A Heart) program on disease-related knowledge, quality of life, and transition experiences in adolescents with CHD. | Pre-posttest study with control group | 28 adolescents and 25 parents in the intervention group; 53 adolescents and 18 parents in the control group, ages ≥12 with moderate to severe CHD | The TWAH program included tailored education, skills training, a transition coordinator, and a joint transfer outpatient visit involving pediatric and adult cardiologists. | There was a significant increase in CHD knowledge (from 59.8% to 75.7%) and improved transition experiences; there was no gap in follow-up for participants. Transition experience and knowledge gains were notably higher in the intervention group compared to the controls. |  |
| Mackie et al. (2019)  Conference proceeding | Canada | Evaluate a transition program for patients with CHD transitioning to adult care. | RCT | Adolescents with CHD | Focuses on the transition from pediatric to adult care services | Improvement in patient adherence and satisfaction |  |
| Mackie et al. (2016)  Journal Article | Canada | To evaluate the impact of a nurse-led transition intervention on the time to first adult congenital heart disease (ACHD) clinic attendance among adolescents with CHD. | Study Protocol | 120 adolescents aged 16–17 with moderate or complex CHD, from two tertiary pediatric cardiology centers in Canada | Two 1-hour individualized nurse-led sessions: Session 1 focused on CHD knowledge and MyHealth Passport creation; Session 2 on self-management skills and role-playing. Conducted ~2 months apart in a pediatric cardiology clinic | Primary outcome: excess time to first ACHD visit. Secondary outcomes: CHD knowledge (MyHeart score), transition readiness (TRAQ, Williams’ scale), and rates of cardiac re-intervention. Study emphasizes treatment fidelity and the clinical relevance of transition preparation. |  |
| Acuña Mora et al. (2017)  Journal Article | Sweden | To assess the effectiveness of a structured, person-centred transition programme in empowering adolescents with CHD during their transition to adulthood. | Hybrid experimental design: RCT embedded in a longitudinal observational study | 210 adolescents with CHD, aged 16 at enrollment; 70 in each arm (intervention, comparison, control); inclusion of parents as secondary participants. | Multicomponent person-centred programme including individualized transition plans, education on CHD, self-management support, peer meetings, and structured transfer to adult care. Intervention delivered over 2 years with 3 outpatient visits, info day, and follow-up. | Study aims to demonstrate increased empowerment (primary outcome) using the GYPES scale. Secondary outcomes: transition readiness, health behaviors, knowledge, quality of life, healthcare use. Results to inform implementation of structured, PCC-based transition models. |  |
| Acuña Mora et al. (2019)  Journal Article | Sweden, Belgium, South Africa | To determine the amount, type, and evidence level of published literature on transfer and/or transition in young people with chronic conditions (CCs), and to describe study characteristics. | Scoping review of published studies | 952 publications included: 790 quantitative, 128 qualitative, 34 mixed/multimethods; majority from North America and Europe | Included studies on transfer/transition of adolescents and young adults (ages 10–25) with chronic conditions; covered various specialties (mostly endocrinology and neurology); majority lacked high-level evidence. | Most studies are low-evidence (levels 4–5), with few RCTs (level 1). Transition research is dominated by certain specialties and regions. Emphasis placed on the need for more experimental studies and inclusion of underrepresented conditions and stakeholders. |  |
| Bassareo et al. (2023)  Journal Article | Ireland | To describe the Irish national model for transition of care in adolescents with congenital heart disease (CHD), within a centralized healthcare system, and propose it as a possible model for Europe. | Descriptive review and implementation model (narrative review) | Not applicable (model-focused article | Transition model includes early start (from age 12), formalized transfer at 16+, multidisciplinary team (PC/ACHD consultants, nurses, psychologists), and structured protocols within a hub-and-spoke system. | The Irish model addresses six key transition areas (advocacy, psychological support, sexual health, autonomy, education, lifestyle). It’s one of the first national models in Europe and meets most ESC transition indicators. Emphasis on early preparation, continuity, and minimizing loss to follow-up. |  |
| Bratt et al. (2018)  Journal Article | Sweden | To explore parents' expectations and needs during their adolescent child's transition to adult care for congenital heart disease. | Qualitative study using semi-structured interviews and content analysis | 18 parents (from 16 families) of adolescents with moderate or complex CHD (ages 13–18), recruited from 4 pediatric cardiology centers | Transition process examined from the parental perspective, focusing on being informed, involved, and supported during the shift from pediatric to adult care. | Two themes: (1) Need for information and involvement to feel secure; (2) Navigating the timing and process of handing over responsibility. Parents emphasized the importance of structured guidance, early and tailored information, inclusion in planning, and assurance that adolescents receive critical CHD-related education. |  |
| Bratt et al. (2022)  Conference Proceeding | Sweden | To investigate the empowering effect of a structured, person-centred transition programme (STEPSTONES) for adolescents with congenital heart disease (CHD), and evaluate its impact on transition readiness, health outcomes, and parental involvement. | Hybrid design: RCT embedded in a longitudinal observational study across 7 Swedish CHD centres | Adolescents with CHD (age 16 at T0, up to 18.5 at T2), randomized in 2 centres (IG and CG), with 5 additional centres as contamination check (CCCG) | Three-assessment points: T0 (16y), T1 (17y), T2 (18.5y); person-centred transition programme delivered in outpatient pediatric cardiology; includes education, empowerment, and support by trained professionals | Empowerment significantly improved in IG vs CG (Δ = 3.44, p = 0.036). Secondary outcomes with significant improvement: parental involvement (p = 0.008), CHD-specific knowledge (p = 0.0002), and satisfaction with physical appearance (p = 0.039). No contamination effect found in CG vs CCCG. |  |
| Bratt et al. (2023)  Journal Article | Sweden | To evaluate the effectiveness of the STEPSTONES person-centered transition program on empowerment (primary outcome) and on transition readiness, health status, knowledge, and parental involvement (secondary outcomes) in adolescents with CHD | Hybrid experimental design: RCT embedded in a longitudinal observational study (3-arm: IG, CG, CCCG) | 208 adolescents with CHD aged 16 at baseline; randomized into IG (70) and CG (69), with 69 in CCCG. Follow-up at 17 and 18.5 years. | Multicomponent, nurse-led intervention (8 elements), delivered over 2.5 years across 5 structured steps, using a person-centered care approach. Included transition planning, peer support, parent guidance, and actual transfer to adult care. | Empowerment improved significantly in IG vs CG (Δ = 3.44; p = .036). Secondary outcomes with significant improvements: CHD knowledge (p = .0002), satisfaction with physical appearance (p = .039), and reduced parental involvement (p = .008). No contamination observed. Supports implementation of structured transition programs in routine care. |  |
| Bredy et al. (2024)  Journal Article | France | To evaluate the impact of a structured transition program on health-related quality of life (HRQoL) in adolescents and young adults with congenital heart disease (CHD). | Multicenter RCT with intention-to-treat and per-protocol analyses | 200 adolescents and young adults with CHD, ages 13–25; randomized to intervention (n=100) or control (n=100) | Holistic transition program including 3 phases: (1) 1:1 educational visit with nurse educator, (2) 1-day group session with multidisciplinary team, (3) joint pediatric-adult cardiology transfer visit. Educational content co-designed with patient advocacy groups. | The program significantly improved self-reported HRQoL (Δ = +3.03; p = .044), psychological HRQoL (Δ = +3.33; p = .049), proxy-reported physical HRQoL (Δ = +9.18; p = .015), and disease knowledge (Δ = +3.13; p < .001). No changes observed in physical/mental health metrics. Supports broader implementation of structured, patient-centered transition models. |  |
| Burström et al. (2016)  Journal Article | Sweden | To identify and describe the needs of adolescents with CHD and their parents during the transition phase before transfer to adult cardiologic care. | Exploratory qualitative study using individual semi-structured interviews and content analysis | 13 adolescents (ages 16–18) with moderate to complex CHD and 12 parents | Interviews explored perceptions about changing roles, information needs, and everyday challenges. Focus on the pre-transfer phase from pediatric to adult cardiology. | Adolescents expressed needs for continuity, responsibility, and adequate knowledge ("safety and control"); parents emphasized trust and a secure handover ("safety and trust"). Both groups valued relationship continuity, clear communication, and individualized, age-appropriate information. Highlights the need for structured, planned transition programs. |  |
| Burström et al. (2019)  Journal Article | Sweden | To assess the level of transition readiness in adolescents with CHD, compare their assessments with those of their parents, and explore correlates of readiness. | Cross-sectional, triadic survey design | 157 triads (adolescent, mother, father); adolescents aged 14–18 with CHD | Used the Readiness for Transition Questionnaire (RTQ) to measure adolescent responsibility, parental involvement, and overall readiness. Multicenter study across four Swedish hospitals. | Adolescents rated themselves as more ready than their parents rated them. Readiness was associated with older age, higher empowerment, and less threatening illness perception. Parental involvement decreased with age. Results underscore the value of triadic perspectives and the role of empowerment in supporting transition readiness. |  |
| Bushee et al. (2021)  Journal Article | USA | To evaluate changes in unplanned cardiac hospitalizations and rates of transfer to adult care after implementing a structured transition program for adolescents with CHD. | Retrospective, single-center comparative cohort study (pre-post design) | 653 patients ≥16 years: 303 in control group (pre-2016) and 350 in transition group (2016–2018) | Transition program included nurse-led education on transition readiness during pediatric visits, yearly follow-ups, and use of a structured readiness assessment. | The transition group had significantly fewer unplanned cardiac hospitalizations (0.004 vs. 0.019 hosp/patient/year; p = 0.008) and higher rates of transfer to adult care (47% vs. 38%; p = 0.034). Being in the transition group was independently associated with reduced hospitalization risk. |  |
| Cabrera Fernandez et al. (2024)  Journal Article | USA and Canada | To examine how social determinants of health (SDoH) impact the transition from pediatric to adult cardiology care in adolescents and young adults with CHD, and to propose strategies to reduce inequities. | Narrative review | Not applicable (review-based) | Focus on SDoH (e.g., race, SES, insurance access, geographic location) affecting CHD care. Recommends multilevel strategies: individual (bias training, education), institutional (formal programs, diversity), and system-level (policy, insurance, telehealth). | Transition is influenced by SDoH like race, SES, and geography. Black, Indigenous, and low-income youth face higher loss to follow-up and poorer outcomes. Strategies to address inequities include early education, standardized transition programs, enhanced insurance coverage, culturally competent care, and workforce diversification. |  |
| Everitt et al. (2017)  Journal Article | USA | To review the current practices, guidelines, barriers, and predictors related to the transition and transfer of care in adolescents and young adults with CHD, and to identify knowledge gaps and quality improvement opportunities. | Narrative review | Not applicable (review-based) | Compares US, Canadian, and European guidelines; highlights structural, institutional, social, and neurocognitive barriers; discusses predictors of successful transition (e.g., self-advocacy, parental involvement, documentation of follow-up need). | Transition programs starting early (age 12–14) and ending by 21 are recommended. Barriers include insurance, institutional aging-out policies, and provider-patient attachment. Predictors of successful transfer include patient beliefs, medical documentation, and prior care continuity. Emphasizes the need for standardized quality indicators, more ACHD-trained providers, and structured programs. |  |
| Fernandes & Landzberg (2004)  Journal Article | USA | To outline the rationale, principles, barriers, and proposed models for effective transition of adolescents and young adults with congenital heart disease (CHD) to life-long adult care. | Narrative review | Not applicable (review-based) | Describes the transition as a planned process requiring education, timing flexibility, and a dedicated transition team; emphasizes need for individualized health passports and coordination between pediatric and adult services. | Highlights barriers (e.g., emotional attachment, lack of adult-trained providers, insurance gaps), recommends transition starting ~17–19 years, and calls for the establishment of regional ACHD centers. Emphasizes the critical role of structured education, insurance counseling, and liaison roles in improving transition success and long-term outcomes. |  |
| Goossens et al. (2014)  Journal Article | Belgium | To evaluate the effectiveness of a structured patient education program on the knowledge level of adolescents and adults with CHD. | Descriptive, cross-sectional comparative study | 317 patients with CHD (226 received structured education, 91 comparison group); age range 15–60 years | Education delivered during ACHD outpatient visits by advanced practice nurses using a standardized checklist; content covered CHD condition, treatment, prevention, lifestyle, heredity, pregnancy, and contraception. | Structured education significantly improved knowledge scores (57% vs. 43%, p < 0.001), but only 11% reached the 80% knowledge target. Education remained an independent predictor of higher knowledge. Calls for alternative or complementary education strategies due to persistent knowledge gaps. |  |
| Habibi (2017)  Journal Article | USA | To describe the transition process for congenital heart patients and propose strategies to prevent loss to follow-up during transfer to adult care. | Descriptive review and expert commentary | Not applicable (review-based) | Emphasizes a structured, nurse-led transition program that begins in early adolescence and continues through young adulthood. Includes education, patient engagement, individualized planning, and interdisciplinary coordination. | Key strategies to prevent loss to follow-up include early transition preparation, dedicated transition coordinators (often CNSs), fostering self-management skills, and ensuring warm hand-offs to adult providers. Highlights the CNS role in assessment, planning, education, and follow-up to support successful transition. |  |
| Harada et al. (2025)  Journal Article | Japan | To review the epidemiology, challenges, and current models of transitional care for adults with congenital heart disease (ACHD), and propose strategies to reduce lapses in care. | Narrative review | Not applicable (review-based) | Advocates for structured, multidisciplinary transition programs starting from age 12, using tools like the Got Transition Six Core Elements and validated questionnaires. Recommends early education, individualized planning, joint clinics, and follow-up post-transfer | Loss to follow-up remains a global issue, with rates up to 34% in the US. Education programs improve knowledge, self-efficacy, and follow-up rates but often fall short of targets. Barriers include low CHD awareness, mild disease, distance, and socioeconomic factors. Highlights the need for tailored interventions, transition coordinators, and digital health tools to improve continuity of care. |  |
| Hayman & Martyn-Nemeth (2022)  Journal Article | USA | To discuss challenges and opportunities in transitioning adolescents and young adults with congenital heart disease to adult-centered care. | Commentary based on literature and American Heart Association statement | Not applicable (review-based) | Advocates for structured transition programs emphasizing self-management, self-advocacy, care coordination, and health literacy. Recommends early initiation in adolescence with continued support through emerging adulthood (18–25 years). | Identifies key barriers (e.g., social determinants, racial/ethnic disparities, neurocognitive deficits). Emphasizes roles of pediatric and adult cardiovascular nurses. Recommends individualized education, lifelong care planning, and system-level changes to support health equity and reduce gaps in care. |  |
| Hays (2015)  Journal Article | USA | To review current recommendations, practices, and gaps in the transition of care for adults with congenital heart disease, particularly in the U.S. context. | Narrative review | Not applicable (review-based) | Emphasizes early, structured transition starting in adolescence and guided by Bethesda and ACC/AHA recommendations. Highlights the role of ACHD regional centers and multidisciplinary teams, and outlines a three-tiered provider education model. | Despite improved survival, U.S. lacks a national transition system. Transition improves outcomes but is inconsistently implemented. ACHD patients have complex psychosocial/medical needs. Nurse practitioners and transition coordinators are key. Calls for more education, research, and advocacy to support comprehensive ACHD transition programs. |  |
| Heery et al. (2015)  Journal Article | Ireland, UK | To synthesize empirical evidence on outcomes and experiences of transition from pediatric to adult healthcare services in young people with congenital heart disease. | Systematic review (13 studies: 7 on outcomes, 7 on experiences) | Studies included various populations from Canada, US, UK, Belgium; sample sizes ranged from 23 to 794 participants. | Reviewed both transfer outcomes (loss to follow-up, lapse in care) and transition experiences (readiness, knowledge, expectations). Included quantitative, qualitative, and mixed methods studies. | High loss to follow-up and care gaps post-transfer are common. Protective factors: belief in need for care, prior surgeries, parent disengagement, and formal referrals. Youth often lacked knowledge and preparation. Structured, personalized transition programs with early education, family support, and trained ACHD professionals are strongly recommended. |  |
| Hummel et al. (2023)  Journal Article | USA | To highlight the importance of integrated care across the lifespan for CHD patients, focusing on effective transition from pediatric to adult care and discussing the example of the Ricci et al. program. | Editorial commentary (with discussion of Ricci et al. 2023 program) | Not applicable (commentary, refers to Ricci et al.'s patient sample) | Describes a nurse-led transition program with patient education, checklists, and longitudinal follow-up, adaptable to virtual delivery during COVID-19. Uses the Transition Service Assessment Questionnaire to identify risks. | The editorial supports Ricci et al.’s model as a reproducible, low-resource intervention that improves CHD knowledge and transition outcomes for most adolescents. However, those with learning disabilities or complex CHD benefit less, requiring more integrated care. Emphasizes the need to measure patient-centered outcomes and integrate services across the care continuum. |  |
| Hwang et al. (2024)  Journal Article | South Korea | To evaluate the effects of an online health management program on self-efficacy, health behavior, and psychosocial outcomes in adolescents with complex CHD during transition to adulthood. | RCT | 28 adolescents aged 12–19 with complex CHD; 14 in intervention group, 14 in control group | 4-week online program based on self-efficacy theory: weekly group sessions (Zoom), 1:1 phone coaching, dietary diary feedback, Facebook-based health info. Covered CHD education, physical activity, diet, stress, and career planning. | Significant improvements in self-efficacy, daily step count, moderate-to-vigorous physical activity, and psychosocial HRQoL in the intervention group. No effect on sleep. High satisfaction and adherence. Suggests online interventions are effective for transition support in complex CHD. |  |
| Jalkut & Allen (2009)  Journal Article | USA | To review the literature on transition from pediatric to adult care in adolescents with CHD and to highlight the role of the pediatric nurse practitioner (PNP) as a transition coordinator. | Narrative review | Not applicable (review-based) | Focus on barriers to transition (e.g., insurance, provider training, family/patient resistance) and role of PNP in facilitating structured education, care planning, and transfer. Emphasizes early initiation (age 11–12), creation of a health passport, and coordinated transition visits. | Transition often fails due to poor education, lack of preparation, and systemic barriers. PNPs can ensure continuity and improve readiness via structured, individualized support. Education, planning, and communication are key to successful transfer to adult CHD care. |  |
| John et al. (2022)  Journal Article | USA (multinational contributors) | To present updated guidance from the American Heart Association on designing and implementing effective transition programs for adolescents with congenital heart disease. | Expert consensus and narrative review | Not applicable (review-based) | Emphasizes individualized, structured programs addressing self-management, self-advocacy, communication, neurocognitive and psychosocial needs. Includes education, family engagement, use of technology, and multidisciplinary support. | SDOH, mental health, neurocognitive deficits, and parental overprotection significantly impact transition. Recommends early initiation, tailored education, and measurement of readiness and outcomes. Nurse-led and tech-based interventions are promising. Advocates for reimbursement strategies and national benchmarks. |  |
| Lee et al. (2024)  Journal Article | South Korea | To evaluate the effectiveness of structured transition programmes for adolescents and young adults with CHD in improving disease knowledge, self-management, and reducing loss to follow-up. | Systematic review and meta-analysis of 10 studies (RCTs, cohort and case-control studies) | 1,297 adolescents and young adults with CHD (ages 10–29), varying CHD severity | Programs included 5 components: transition intro, medical knowledge, living with CHD, self-management, and self-advocacy. Delivered by nurses/teams in individual or group formats. Parental involvement in some studies. | Transition programmes significantly improved disease knowledge (Hedges’ g = 0.89), self-management (g = 0.67), and reduced loss to follow-up (OR = 0.41). No significant effect on quality of life. Evidence quality was low to very low; further research needed. |  |
| Lopez et al. (2024)  Journal Article | USA | To describe social, demographic, and clinical factors associated with successful transition of care in adolescents with CHD at a tertiary urban center. | Retrospective, single-center cohort study (chart review 2015–2021) | 322 patients aged ≥15 seen in pediatric cardiology (2013–2014); 49% transitioned, 22% continued in pediatric care, 29% lost to follow-up | Transition success defined as at least one adult CHD/cardiology visit. Predictors analyzed: CHD severity, insurance, defibrillator presence, visit frequency, meds. No structured transition program was in place. | Successful transition associated with more complex CHD (p=0.0002), insurance coverage (p<.0001), presence of defibrillator (p=0.0028), frequent pediatric visits (p=0.0005), and more medications. Patients with simple CHD and no insurance were more likely to be lost to follow-up. Calls for formal, structured transition programs. |  |
| Mackie et al. (2014)  Journal Article | Canada | To evaluate the impact of a 1-hour nurse-led transition intervention on self-management, self-advocacy, and cardiac knowledge in adolescents with moderate or complex CHD or cardiomyopathy. | Clinical trial with systematic allocation (by clinic week) | 58 adolescents aged 15–17 years (27 intervention, 31 control); most had moderate/complex CHD | One-time, 1-hour individualized nurse-led session including CHD education, MyHealth Passport creation, discussion of complications, and lifestyle topics. TRAQ and MyHeart scores assessed at baseline, 1 and 6 months. | At 6 months, the intervention group had significantly higher self-management (TRAQ: 3.59 vs. 3.16, p = .048) and CHD knowledge scores (MyHeart: 75% vs. 61%, p = .019) than controls. Suggests brief, structured education improves transition readiness and disease understanding. |  |
| Moons et al. (2008)  Journal Article | Belgium | To discuss the scope and consequences of loss to follow-up in CHD patients and to argue for the implementation of transition programs to prevent it. | Narrative review and expert commentary with supporting data from multiple studies | Not applicable (references to studies from Germany, USA, Canada, Belgium) | Emphasizes the need for structured transition programs including education, navigation skills, early preparation, and timely transfer from pediatric to adult CHD care. | 50–75% of CHD patients are lost to follow-up, including those with complex lesions. Loss to care is linked to significant morbidity and increased risk for urgent interventions. Transition programs can prevent a new "lost generation" by ensuring continuity of care, especially through education and system navigation. |  |
| Moons et al. (2009)  Journal Article | Belgium | To explore the expectations and experiences of adolescents with CHD regarding their transfer from pediatric cardiology to an adult congenital heart disease (ACHD) program. | Qualitative, phenomenologic study using semi-structured interviews | 14 adolescents aged 15–17 years with CHD (various severity), recently transferred or scheduled for transfer | Transfer to ACHD program at age 16; no formal transition program; interviews explored attitudes toward transition, information needs, and shifting roles between adolescents and parents. | Six themes emerged: transition viewed as normal; loss of familiar environment; curiosity/positive attitude toward ACHD care; adjustment challenges; lack of information; evolving independence vs continued parental support. Highlights need for structured transition planning and adolescent-focused communication. |  |
| Moons et al. (2021)  Journal Article | International (multi-organization) | To provide global consensus recommendations on the transition and transfer of adolescents with CHD to adult care, tailored to different resource settings. | Expert consensus (position paper by international societies) | Not applicable (review-based) | Recommends a structured, individualized transition process beginning at age 12, including assessment, education, peer support, empowerment, and eventual transfer to ACHD care. Advocates the transition coordinator model as preferred. | Emphasizes lifelong follow-up, prevention of care gaps, and patient empowerment. Highlights different models for transition (e.g., coordinator, joint clinic) and outlines key steps from pre-transition to post-transfer. Recognizes variability in global resources but advocates for transition planning in all settings. |  |
| Moons et al. (2023)  Journal Article | Belgium | To advocate for empowerment as a key outcome of transition programs in adolescents with CHD and to discuss methodologies for designing effective interventions. | Expert editorial commentary based on evidence synthesis | Not applicable | Emphasizes structured, individualized transition programs incorporating education, support, empowerment, and co-design with patients and families. Highlights the STEPSTONES program and intervention mapping as model approaches. | Empowerment is a practical and impactful outcome of transition care, influencing QoL and health behaviors. Co-designed programs, including peer support, tailored education, and involvement of nurses and social workers, can enhance self-management and patient engagement in CHD care. |  |
| Moons et al. (2025)  Journal Article | Belgium | To synthesize current evidence on transitional care for adolescents with CHD and argue for its formal recommendation as Class I, Level A. | Expert commentary based on multiple RCTs and consensus guidelines | Not applicable | Reviews structured transition programs (e.g., CHAPTER and STEPSTONES) with nurse-led education, empowerment focus, and support components across adolescence. | Two recent RCTs (STEPSTONES in Sweden, TRANSITION-CHD in France) demonstrate improvements in empowerment, CHD knowledge, HRQoL, and reduced care gaps. Based on this, transition programs now qualify as Class I, Level A recommendation in CHD care. |  |
| Murphy & Foster (2005)  Journal Article | USA | To provide training guidelines for pediatric cardiologists on the transition of care and management of adults with CHD. | Expert consensus (Bethesda Conference Task Force 6) | Not applicable | Emphasizes structured training at three levels (core to advanced) for pediatric cardiologists. Recommends preparing adolescents for transition, understanding adult CHD outcomes, and promoting liaison between pediatric and ACHD services. | Highlights the need for transition preparation, inclusion of adolescent and adult health issues in pediatric training, and collaboration between pediatric and adult cardiologists. Supports specialized training to improve continuity and quality of CHD care during transition to adulthood. |  |
| Nicolarsen (2017)  Journal Article | USA | To review challenges, progress, and future directions in the transition of adolescents and young adults with congenital heart disease. | Narrative review and expert opinion | Not applicable | Discusses differences between pediatric and adult care models; emphasizes the need for structured transition programs with assessment tools, education, psychosocial support, and communication between providers. | Gaps in care are common (e.g., 42% had >3 years without follow-up). Transition programs improve continuity, but lack outcome data. Barriers include cognitive/social issues, lack of provider awareness, and access to ACHD centers. Calls for early transition preparation, structured programs, and more trained specialists. |  |
| Niwa (2015)  Journal Article | Japan | To provide an overview of issues and recommendations related to the transition of adolescents with congenital heart disease to adult care. | Narrative review | Not applicable | Emphasizes structured, multidisciplinary transition programs that begin in early adolescence and continue into adulthood. Advocates for the involvement of ACHD specialists, nurses, and mental health professionals. | Highlights challenges, including patient non-compliance, cognitive deficits, and health system barriers. Recommends transition coordinators, educational strategies, and international collaboration to improve outcomes. Stresses that early, continuous, and patient-centered transition is essential to reduce morbidity and mortality. |  |
| Saarijärvi et al. (2021)  Journal Article | Sweden | To explore adolescents' and parents' experiences of participating in the STEPSTONES person-centred transition programme and identify mechanisms of impact leading to empowerment. | Qualitative study embedded in an RCT (process evaluation) | 14 adolescents and 12 parents from the intervention group of the STEPSTONES trial | Programme included a transition coordinator, goal setting, CHD education, written plan, peer support, and contact with adult care over 2.5 years. Person-centred approach with emphasis on empowerment. | Participants reported increased empowerment through safe and personalized support, improved CHD knowledge, communication skills, and readiness for transfer. Key mechanisms: continuity, confidentiality, peer support, and tailored education. Some parents felt insufficiently involved. Findings support implementation of structured, multi-component transition programmes. |  |
| Saarijärvi et al. (2022)  Journal Article | Sweden | To evaluate the implementation fidelity of the STEPSTONES transition program for adolescents with CHD, and to identify moderating factors affecting program delivery. | Mixed methods process evaluation embedded in an RCT | 59 adolescents with CHD (from 67 randomized), 2 transition coordinators, 4 ACHD nurses | STEPSTONES: 8 components over 2.5 years, delivered in pediatric outpatient settings by trained transition coordinators using person-centred care and behavior-change techniques. Included individual visits, goal setting, education, parent guidance, peer support, and joint transfer visit. | High fidelity in 6/8 components; peer support had low attendance (32.2%) and transfer meeting had implementation challenges. Moderating factors included adolescent maturity, engagement, contextual constraints (e.g., lack of SOPs), and coordination issues between pediatric and adult care. Emphasizes balance between fidelity and local tailoring for implementation success. |  |
| Sable et al. (2011)  Journal Article | USA | To provide best practice recommendations for managing transition to adulthood for adolescents with CHD, covering medical, psychosocial, and systemic aspects. | Expert consensus (Scientific Statement by AHA) | Not applicable | Recommends a structured, individualized transition process starting by age 12, including education, self-management, family support, and coordinated transfer to ACHD care. Highlights the role of transition coordinators, health passports, and assessment tools. | Poorly planned transitions result in care gaps and adverse outcomes. Early, flexible, and developmentally appropriate interventions improve readiness, QoL, and follow-up. Multidisciplinary teams, primary care collaboration, and policy changes are essential. Lists specific recommendations for adolescents, parents, providers, and systems of care. |  |
| Said et al. (2015)  Journal Article | USA | To review the challenges and key elements of transitioning care for adolescents with congenital heart disease from pediatric to adult healthcare providers. | Narrative review | Not applicable | Emphasizes early, individualized transition starting around age 12; includes education, coordinated care, psychosocial support, planning for insurance, and transfer to ACHD-trained providers. | Transition often disrupted by lack of provider training, institutional constraints, and insurance loss. Successful transition depends on structured programs, dedicated teams, continued education, and specialized adult CHD centers. Late complications require lifelong surveillance, and early mortality is reduced when congenital-trained surgeons are involved. |  |
| Skogby et al. (2021)  Journal Article | Sweden | To explore young adults’ perceptions and experiences of factors influencing continued follow-up care after transition from pediatric to adult congenital heart disease (CHD) services. | Qualitative descriptive study with content analysis of individual interviews | 16 young adults with CHD (13 in follow-up, 3 without), aged 27–29; recruited from all 7 Swedish university hospitals | Explored factors influencing follow-up attendance post-transfer. Focused on motivation, relationship with healthcare providers (HCPs), and care accessibility in a low-discontinuation setting. | Continued care is multifactorial: key facilitators included CHD knowledge, clear follow-up purpose, supportive HCP relationships, accessible and flexible services. Barriers included low risk perception, parental overinvolvement, anxiety, and systemic issues like travel and cost. Emphasizes holistic, person-centered strategies to prevent care discontinuation. |  |
| Talluto (2018)  Journal Article | USA | To review key components and barriers to establishing a successful transition program for adolescents with congenital heart disease. | Narrative review | Not applicable | Emphasizes the difference between “transition” and “transfer”; structured transition should begin at age 12 with a written plan by 14; includes education, self-management, readiness assessment, and joint pediatric-adult collaboration. | Barriers exist at pediatric, patient/family, and adult system levels. Structured transition programs improve continuity, reduce loss to follow-up, and must include written policies, trained coordinators, and institutional support. Calls for more research on best practices and outcome measures for successful transition. |  |
| Thomet et al. (2015)  Journal Article | Switzerland | To review models, challenges, and best practices in transition from pediatric to adult care for adolescents with congenital heart disease, drawing from the experience of two Swiss centers. | Narrative review with comparative program descriptions | Not applicable | Comparison of two structured transition models: nurse-led (Bern) and physician-led (Zurich). Both involve early education (~12–14 years), patient/family engagement, and collaboration between pediatric and adult CHD services. | Effective transition requires early, individualized, and multidisciplinary planning. Adolescents, parents, and providers all play key roles. Structured programs (especially with transition coordinators) prevent care gaps. Transition should not be confused with transfer and must continue post-handover until self-management is achieved. |  |
| Thomet et al. (2021)  Journal Article | Europe (24 countries) | To provide a contemporary view of transfer and transition practices for adolescents with CHD across European ACHD centres, and evaluate alignment with quality indicators. | Cross-sectional descriptive survey of ACHD centres | 96 ACHD centres meeting criteria (≥1 ACHD-trained cardiologist; ≥200 ACHD patients in follow-up) | 41.7% had formal transition programmes; 88.5% had structured transfer processes. Transition often nurse-led, beginning ~age 12–16. Multidisciplinary teams involved. Indicators assessed: timing, flexibility, documentation, education, and coordination. | Only 4 centres met all quality indicators; 31% met at least 4 for both transfer and transition. Key gaps: lack of written protocols, limited flexibility, incomplete coverage. Larger centres more likely to offer formal transition. Nurses play a central role. Emphasis on need for individualized, developmentally appropriate transition beyond formal transfer. |  |
| Thomet et al. (2023)  Journal Article | Switzerland | To develop and validate a set of evidence-based quality indicators (QIs) for transfer and transition programmes in adolescents with CHD. | RAND/UCLA modified Delphi process with literature review and expert panel | 16 international experts (clinicians, researchers, patients) in CHD and transition care | 12 QIs (5 structure, 7 process) were finalized, covering components like transition policy, coordinator, education, peer support, confidentiality, and handover; based on literature and expert consensus. | This is the first set of structured QIs for CHD transition care. The QIs are feasible, relevant, and suitable for benchmarking. Further testing in clinical practice is needed. Highlights importance of person-centred care, early preparation, and individualized support in transitional care |  |
| Tyagi & Sontakke (2023)  Journal Article | India | To explore the significance of timely transition for children with CHD to adult care and outline a multidisciplinary approach for successful transition. | Narrative review | Not applicable | Describes a structured, phased transition model (pre-transition, transition, post-transition) starting at age 12. Emphasizes multidisciplinary care, education, self-management skills, peer contact, and sexual health counseling for girls. Advocates personalized plans and monitoring tools like TRAQ. | Emphasizes early, structured transition supported by nurses, psychologists, and other professionals. Highlights importance of patient empowerment, continuity of care, and role of families. Underscores the need for global implementation of QIs and telemedicine to reduce care gaps and improve outcomes. |  |
| Van Deyk et al. (2004)  Journal Article | Belgium | To examine educational and behavioral challenges in transitioning adolescents with CHD from pediatric to adult-centered care and propose strategies for effective patient education. | Narrative review | Not applicable | Emphasizes a structured educational program tailored to adolescents’ developmental stages, covering CHD knowledge, endocarditis prevention, reproduction, lifestyle, and psychosocial aspects. Advocates nurse-led individualized education and early engagement of patients. | Significant knowledge gaps exist in both parents and adolescents regarding CHD, medications, endocarditis, and risk management. Structured, repeated, and developmentally appropriate education—led by advanced practice nurses—is key to successful transition and self-management. |  |
| Vonder Muhll (2020)  Journal Article | Canada | To reflect on global progress in transition from pediatric to adult care for adolescents with CHD and examine system-level predictors of successful transfer. | Editorial commentary with synthesis of empirical studies | Not applicable | Discusses various models (structured programs vs. simple transfer letters); highlights international differences based on health system architecture, insurance coverage, and program volume. | Successful transition is driven not only by patient factors but also by system-level elements: universal health insurance, co-located pediatric/adult programs, shared medical records, and administrative coordination. Sweden and Belgium show high follow-up retention. Interventions must go beyond patient education to structural reforms. |  |
| Werner et al. (2021)  Journal Article | France | To assess the impact of a structured transition education program on health-related quality of life (HRQoL) in adolescents and young adults with CHD. | Study protocol for RCT | 200 CHD patients aged 13–25 years (100 per arm), recruited in 3 tertiary centers | Three-part program: (1) individual educational visit with a nurse; (2) 1-day group session with a multidisciplinary team; (3) joint pediatric–adult cardiology visit. Covers CHD knowledge, self-efficacy, psychosocial issues, and administrative guidance. | Primary outcome: change in HRQoL at 12 months (PedsQL). Secondary outcomes: clinical data, knowledge, exercise capacity, psychosocial status. Hypothesis: education will improve HRQoL by 7±13.5 points. Qualitative analysis will assess participant experiences and program acceptability. Results pending. |  |
| Williams (2015)  Journal Article | USA | To summarize challenges, successes, and recommendations related to the transition of youth with congenital heart disease to adult healthcare. | Narrative review | Not applicable | Emphasizes transition as a process involving preparation, transfer, and continuity of care. Recommends early, continuous education; integration of pediatric and adult systems; and individualized approaches. | High loss to follow-up globally. Key barriers: lack of preparation, low health literacy, cognitive deficits, psychosocial issues, and insufficient ACHD workforce. Successful systems (e.g., Belgium) use integrated records, nurse-led education, and proximity of services. Calls for systemic change, including more ACHD centers, formal training, and personalized transition planning. |  |
| Yamamura et al. (2022)  Journal Article | Japan | To review the principles and practice of transitional care in pediatric cardiology and propose structured approaches to improve continuity for adolescents with CHD transitioning to adult care. | Narrative review | Not applicable | Emphasizes early, individualized education (starting from age 4–7), stepwise shift in responsibility, coordination of medical and social/welfare transitions, and multidisciplinary collaboration. Uses tools like readiness checklists and tailored communication. | Japan has begun developing guidelines and tools, but specialized transitional care remains limited. Early, repeated education is key. System-level differences between pediatric and adult care must be addressed. Cardiologists should lead cross-specialty collaboration. Calls for the integration of social services and reproductive counseling into transition planning. |  |
| Yap et al. (2023)  Journal Article | UK | To evaluate the effectiveness of the Cambridge cardiac transition pathway in achieving successful transfer to adult CHD care. | Retrospective observational study | 179 adolescents with CHD (ages 15–19) seen in the joint transition clinic (2009–2018); 142 eligible for analysis | Structured, joint pediatric-adult CHD transition clinic with nurse and psychologist support, pre-transfer assessment, separate meetings, education on lifestyle, contraception, and planning. Reminders used to ensure attendance. | 98.6% successfully transferred; 78% attended first adult visit directly, 20% after reminders, and only 1.4% failed. Key factors: joint clinics, early planning, involvement of adult team, nurse-led coordination. Model deemed effective and replicable. |  |
| Legend:  CHD: Congenital Heart Disease; IG: Intervention Group; CG: Control Group; CCCG: Contamination Check Control Group; RCT: Randomized Controlled Trial; PROs: Patient-Reported Outcomes; RI-CLPM: Random-Intercept Cross-Lagged Panel Model; T0: Baseline time point (start of the study); T1: Follow-up time point (first follow-up); T2: Final follow-up time point; LLCC: Lifelong Cardiac Care; ACHD: Adult Congenital Heart Disease; TRAQ: Transition Readiness Assessment Questionnaire; AAP: American Academy of Pediatrics; ACP: American College of Physicians | | | | | | | |
