# Supplementary material for: Bridging Pediatric to Adult Care: A Scoping Review on Transitional Care for Individuals with Congenital Heart Disease Using Data Mining Techniques to Identify Key Topics
Source: Curr Cardiol Rep. 2026 May 22;28(1):57. doi: 10.1007/s11886-026-02377-1 (PMC13194312; doi:10.1007/s11886-026-02377-1)
Supplement: Supplementary file 3 — (DOCX 58.3 KB) [file 11886_2026_2377_MOESM3_ESM.docx]

**Supplementary File 3**. Additional information on LDA

The process began with data preprocessing. After loading the data from an Excel file containing key content, we created a text corpus using the tm package. Several preprocessing steps were applied to clean the data: the text was converted to lowercase, punctuation and numbers were removed, and common English stopwords were eliminated. We then stripped excess whitespace and stemmed the words to reduce them to their root forms, ensuring a consistent input for analysis.

With a clean corpus, we proceeded to construct a Document-Term Matrix (DTM), which provided the structured input required for topic modeling. To determine the optimal number of topics, we used the FindTopicsNumber function from the ldatuning package, testing models with topics ranging from 2 to 8. Four evaluation metrics guided the selection of the ideal topic count: the Griffiths2004 metric, which should be maximized; the CaoJuan2009 and Arun2010 metrics, both to be minimized; and the Deveaud2014 metric, another to be maximized. Additionally, a Bayesian optimization was performed to refine the selection process and confirm the robustness of our results. This optimization process indicated that three topics, as suggested by the metrics, were the optimal solution for this dataset. We applied the Gibbs sampling method to train the models, setting a random seed for reproducibility.

The results were visualized in a plot that shows how each metric varied across different numbers of topics. The top panel of the plot highlights the metrics to be minimized (CaoJuan2009 and Arun2010), while the bottom panel displays the metrics that should be maximized (Griffiths2004 and Deveaud2014). This visualization allowed for an intuitive selection of the optimal number of topics by observing points where the metrics reach minimal or maximal values, suggesting the most coherent topic structure for the dataset. The solution extracting 4 topics resulted in the optimal one.


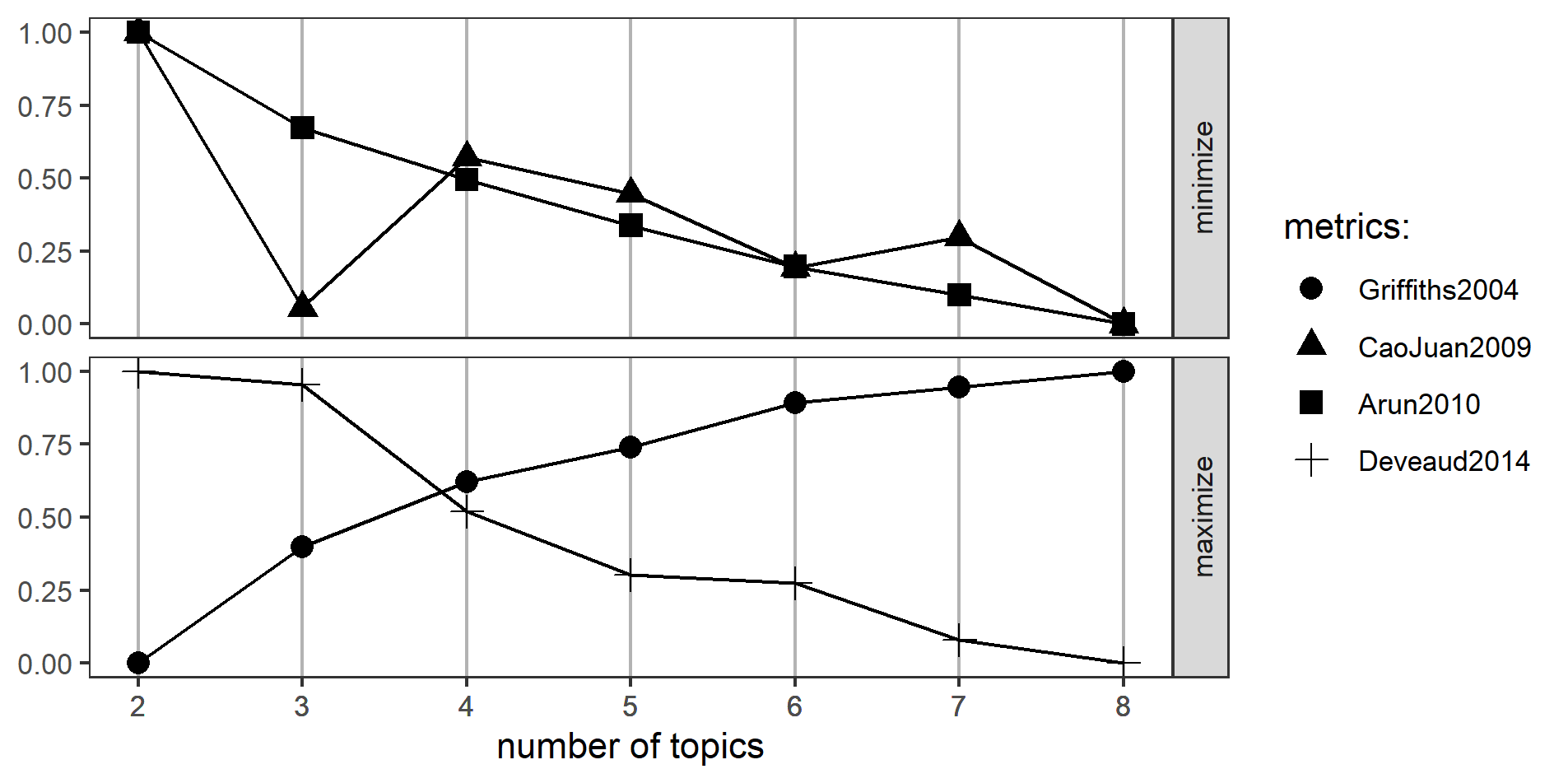


To further analyze the content and themes within our dataset, we conducted Latent Dirichlet Allocation (LDA) with four topics, which was determined to be the optimal number based on Bayesian optimization and various metrics. Below is a detailed explanation of the steps and output obtained:

After setting the number of topics to four, we applied LDA modeling to our Document-Term Matrix (DTM). Using the tidytext package, we extracted the beta matrix, which represents the probability of each term appearing in each topic. To ensure readability, we truncated the terms to 15 characters.

For each topic, we selected the terms with the highest beta values (probabilities), which indicate the most representative words for that topic. This enabled us to identify distinguishing themes within each topic based on prominent terms.

To visualize the themes associated with each topic, we generated word clouds for each one. The function plot_and_save_wordcloud was defined to create and save these visualizations, which display the most frequent terms for each topic with a scaled representation of their probabilities. These word clouds, saved as high-resolution JPEG images, offer a quick overview of the top terms characterizing each topic.

Finally, we listed the top 20 terms for each topic, sorted by beta values, providing a detailed view of the key terms. This table allows for a more granular inspection of each topic's thematic structure. For instance, topic 1 includes terms such as “group,” “intervent,” and “patient,” while topic 2 highlights terms like “readi,” “transit,” and “adolesc.” Each topic thus reveals specific areas of focus within the dataset.

The output provided below lists the top 20 terms for each of the four topics, with their respective beta values. This summary reflects the prominent themes within each topic, as identified by the LDA model:

# Groups: topic [3]

topic term beta

*<int>* *<chr>* *<dbl>*

1 1 transit 0.0460

2 1 program 0.0434

3 1 chd 0.0262

4 1 includ 0.0220

5 1 care 0.0202

6 1 adolesc 0.0189

7 1 educ 0.0163

8 1 intervent 0.0124

9 1 parent 0.0123

10 1 success 0.0122

11 1 followup 0.0121

12 1 support 0.0117

13 1 plan 0.0116

14 1 coordin 0.0113

15 1 effect 0.0104

16 1 evalu 0.0103

17 1 structur 0.00983

18 1 health 0.00909

19 1 continu 0.00874

20 1 signific 0.00864

21 2 care 0.0353

22 2 structur 0.0285

23 2 educ 0.0265

24 2 adult 0.0227

25 2 need 0.0186

26 2 transit 0.0180

27 2 improv 0.0168

28 2 followup 0.0167

29 2 health 0.0163

30 2 knowledg 0.0144

31 2 assess 0.0143

32 2 support 0.0134

33 2 diseas 0.0133

34 2 readi 0.0129

35 2 prepar 0.0109

36 2 chd 0.0106

37 2 focus 0.00994

38 2 involv 0.00938

39 2 selfmanag 0.00922

40 2 outcom 0.00922

41 3 transit 0.0938

42 3 chd 0.0344

43 3 adolesc 0.0330

44 3 adult 0.0255

45 3 knowledg 0.0236

46 3 transfer 0.0196

47 3 individu 0.0155

48 3 improv 0.0151

49 3 care 0.0136

50 3 pediatr 0.0134

51 3 educ 0.0130

52 3 readi 0.0115

53 3 earli 0.0110

54 3 outcom 0.00962

55 3 intervent 0.00950

56 3 achd 0.00910

57 3 structur 0.00800

58 3 involv 0.00787

59 3 session 0.00780

60 3 provid 0.00754

The labels for each topic were identified through a qualitative interpretation of the top terms associated with each topic, as revealed by the LDA model. By examining the 20 most probable words in each topic, we could infer the main themes represented and formulate descriptive labels that accurately encapsulate the content. Here’s a breakdown of the reasoning behind each label:

🟦 Topic 1 – "Transition Program Implementation and Coordination"

Keywords: transition, program, care, adolescents, coordinator, intervention, plan, support, evaluation, success

Description:

This topic focuses on how transition programs are designed and coordinated, including structural elements (e.g., plan, structure, evaluation), key professional figures (coordinator, support), and expected outcomes (success, effect, follow-up). The emphasis is on the concrete implementation of programs and their operational impact.

🟩 Topic 2 – "Education, Self-Management, and Structured Support"

Keywords: structured, education, adult, readiness, self-management, support, assessment, knowledge, improvement, focus

Description:

This topic centers on patient empowerment through education and skill development. It emphasizes educational interventions (education, knowledge), assessment tools (assessment, readiness), and support for autonomous health management (self-management, support), all embedded within structured care contexts.

🟧 Topic 3 – "Timing, Knowledge Transfer, and Developmental Needs"

Keywords: transition, adolescents, knowledge, pediatric, adult, ACHD, provider, early, transfer, outcomes

Description:

This topic highlights the temporal and cognitive dimensions of the transition process, with emphasis on early preparation, CHD knowledge, and the roles of healthcare providers. It focuses on the developmental needs of adolescents and the continuity of information and relationships across pediatric and adult care settings.
